# Assessing self–other agreement and dyadic adjustment in marital dyads

**DOI:** 10.3389/fpsyg.2024.1363165

**Published:** 2024-11-15

**Authors:** Joshua D. Dwire, Marvin W. Acklin

**Affiliations:** ^1^Independent Practice, North Las Vegas, NV, United States; ^2^Department of Psychiatry, John A. Burns School of Medicine, University of Hawaii, Honolulu, HI, United States

**Keywords:** couple psychology, marital adjustment, cross-informant assessment, divorce psychology, inter-parental conflict

## Abstract

In this study, we examined self–other agreement in married couples to examine the association between their perceptions of self and other psychological problems and dyadic adjustment. We also postulated that dyadic adjustment would moderate self–other agreement ratings on low- and high-visibility traits of psychological problems. Using a cross-informant assessment design, 101 married dyads in three marital groups (non-clinical, transplant, and divorcing dyads) provided reciprocal self and other ratings for psychological problems. Self–other agreement indices were quantified using self–other differences scores and Pearson *r,* qualifying (*Q*) correlations. The self–other agreement models yielded significant differences in dyadic adjustment across couple types. Couples that demonstrated moderate to elevated levels of self–other agreement for psychological problems had higher levels of dyadic adjustment. Differences in self–other psychological problem ratings were robust predictors of dyadic maladjustment and poor relational quality. Dyadic adjustment was found to moderate self–other agreement for psychological problems, especially for wives’ appraisals and husbands’ attunement to wives’ low-visibility problems. The findings validate the impact of self–other agreement in models of relationship conflict and adjustment. Wive’s other views tended to have large effect sizes in dyadic adjustment. The study’s limitations and recommendations for future research are also discussed.

## Introduction

The way that spouses view themselves and their partners is central to relationship quality and adjustment. Spouses in disagreement over self and other views will negatively alter the perceived quality of the marital relationship system, which requires adjustments of both spouses to maintain—dyadic adjustment. Concordance in married couples’ perception ratings of each other is significantly correlated with marital adjustment and subsequent satisfaction (Murstein and Beck, 1972). Relationship adjustment is an interactional system between the perceiver, the target of the perception, and the perceptions of relationship quality for better or worse impact on the accuracy of the ratings (Funder, 1995). Discrepancies in self and other agreements are potential clinical indicators of dyadic conflict (Grills and Ollendick, 2002) and predict negative outcomes or behavioral problems (Tackett, 2001). Dyadic conflict increases the potential for problems because the way that spouses perceive themselves and each other is reciprocally affected by relationship adjustment and the perceived resulting quality of the relationship.

As social creatures, people interact with others as others simultaneously interact with them. Social and personality psychology has postulated that the sense of self is defined and experienced through reciprocal interactions with others (Mead, 1934). An individual’s self-concept may be viewed as a dynamic synthesis of self and other appraisals (Erikson, 1995; Myers, 2005) as they are interdependently formed by self-monitoring behaviors in relation to the actual and perceived appraisals of others (Cooley, 1902; Oltmanns et al., 2005). Symbolic interactionism (Mead, 1934) described individual perceptions as based upon indirect representations of a particular social group member or the generalized interpretations of the whole social group (Shrauger and Schoeneman, 1979; Kenny and DePaulo, 1993). The self is, therefore, a psychological manifestation of the way social interaction is organized and given meaning by individuals (Ryan et al., 1991).

Self–other agreement studies in social and personality psychology focus on inter-personal perception within familiar relationships—social perceptions, the way people see themselves and the way they are seen by others (Funder and West, 1993; Funder, 1995). Cronbach (1955) noted the “distressingly low consistency of scores…chiefly concerned with differences, perceivers either in terms of their accuracy or in terms of their tendency to view others as similar to themselves” (p. 177). He laid the foundation for a new realm of psychometric studies of self and other agreements with warnings about the mathematical complexity of self–other ratings, noting that “social perception scores” were complex aggregates of factors.

Accuracy of personality judgments within interpersonal perception was defined as consensus of self–other agreement (Funder and West, 1993). Accuracy agreement between judges represented the convergence of trait judgments within self–other ratings (Funder and West, 1993; Watson et al., 2000a). Self–other agreement demonstrated only modest consensus, even in dyads that knew each other well (Cronbach, 1955; Funder and West, 1993). Factors that enhance or detract from self–other agreement have been examined including trait visibility (Funder and Dobroth, 1987) and acquaintanceship (Funder and Colvin, 1988; Kolar et al., 1996). Inter-judge agreement is the highest for visible personality traits that are subject to repeatedly observed behaviors. Habitual expression of observable behavior is easily confirmed and necessary to establish a trait recognition pattern (Funder and Dobroth, 1987).

In contrast to highly visible behaviors, self–other agreement for internal subjective states (i.e., fear, hostility, guilt, sadness, shyness, fatigue, and surprise) was examined in self and peer ratings (Clark and Watson, 1991; Watson and Clark, 1991). Modest but significant self–peer agreement was discovered across affect scales (*r* ranging from 0.19 to 0.41). Self–peer agreement was enhanced with the addition of peer raters and greater judge–target interpersonal knowledge. Highly visible personality traits produced higher inter-judge agreement correlations than low visibility of internalized subjective traits (Watson and Clark, 1991). Trait visibility for Big Five personality factors examined in married, dating, and friendship dyads yielded significant effects ranging from 0.19 to 0.61. Deep interpersonal knowledge and prevalent socially favorable traits elicited higher levels of inter-judge agreement, although no moderating effects of self–other agreement were revealed (Watson et al., 2000a, 2000b).

Perceptions of relationship quality between judge and target are expected to moderate the accuracy of self–other judgment as a poor opinion of someone tends to reduce the accuracy of judgments about the person (Funder, 1995). Causes for disagreement between self-reports and spouse ratings of personality have been examined in a sample of 91 well-acquainted couples (McCrae et al., 1998). Sources of disagreements were self-biases, other-acquiescence, unconscious denial, and faking bad. Moderating effects of relationship characteristics were not identified (McCrae et al., 1998). Personality trait influence on self-spouse agreement for maladaptive personality features was discovered to have a moderate agreement between self and spouse reports of maladaptive personality traits ranging from 0.21 to 0.47 with mean *r* = 0.36 (South et al., 2011).

Coefficients of similarity within spousal perceptions were found to be significantly correlated with marital adjustment in a study of 60 married couples on the Locke–Wallace Marriage Adjustment Scale (Murstein and Beck, 1972). A meta-analysis of 119 studies of cross-informant correspondence in reports of children’s mental health found correspondence levels exhibited a range from low to moderate in magnitude (Achenbach and Edelbrock, 1978). Meta-analysis of 341 available studies, which investigated correspondence among parent, teacher, and child reports, corroborated the previous findings of Achenbach. The results suggested an overall cross-informant correlation of 0.28, with larger correspondences for highly visible (externalized) than lower visibility (internalized) concerns (De Los Reyes et al., 2015). These studies are directly relevant to cross-informant assessment of adult mental health and relationships, even though they originated in a child-based population. These findings highlight contextual features in cross-informant assessment, including concerns about correspondence differences and greater correspondence for externalizing versus internalizing problems.

Differences in parent–child ratings on psychological problems (psychopathology) are associated with relationship conflict, and ratings may be subject to attribution biases (De Los Reyes and Kazdin, 2004, 2005). A framework for understanding how biases can affect actor and observer ratings suggests that differences may be integrally related to dysfunctional interactions between informants based upon different perceptions of the target person, their behavior, the relational interaction between target and rater, or negative reactions to any disparities between the observations (ABC Model; De Los Reyes and Kazdin, 2005). Standardized difference scores were deemed the measure of choice to examine dysfunctional interaction (De Los Reyes and Kazdin, 2006). Familial conflict (dysfunctional interactions) is a crucial factor causing parental disagreement regarding child behavior ratings (Grills and Ollendick, 2002). These interaction factors are also expected to apply to self–other attributions in adult relationships.

### Theory

We theorize that differences in self–other agreement on psychological problem ratings influence dyadic adjustment and are the basis for relationship conflict. Alternatively, relationship adjustment may moderate consensus in self–other appraisals. A state of cognitive dissonance (Harmon-Jones and Mills, 1999) occurs when spouses receive incongruent feedback from each other. Cognitive dissonance is associated with an internalized negative affect state in the perceiver. As an unpleasant affective state, various strategies are utilized to manage or resolve dissonance, including blaming, labeling, minimizing, and so forth (Festinger, 1962). When spousal appraisals are different from the receiver’s self-appraisals, this discrepancy is often reflected in ratings of other psychological problems. In conflictual dyads, these appraisals may reflect negative biases, fundamental attribution errors, and potential self-fulfilling prophecies (Merton, 1968).

### Purpose

This study tested two models of marital interaction by examining similarities and differences in dyadic psychological problem ratings and associations with relationship quality. The study utilized two indices for assessing self–other agreement: difference scores and qualifying similarity coefficients. The self–other agreement approach was applied to utilize empirically validated assessment measures in testing a model suggesting that differences in self–other agreement regarding psychological problems can predict dyadic adjustment. Three groups of married couples (divorce, liver transplant, and non-clinical couples) were chosen to demonstrate a broad range of variance in dyadic adjustment. The study of dyads with deep interpersonal knowledge in marital relationships is essential, considering that partners are dynamic, altering their behaviors in response to the relational interdependence and mutual influence between spouses (Reis et al., 2002). Two alternative models were assessed: the use of self–other difference scores as predictors of dyadic adjustment in relationship quality and relationship quality as a moderator of self–other agreement. A cross-informant assessment model was required to reciprocally examine independent and interaction effects from self and other ratings on psychological problems with a measure of relationship adjustment indicating marital quality.

### Research questions

1. Do different groups of couples differ on the level of relationship adjustment?
2. Do different groups of couples differ on indices of self–other agreement for overall psychological problems?
3. Do different groups of couples differ on ratings of self–other agreement for low- and high-visibility traits of psychological problems?
4. Do self–other agreement ratings of psychological problems predict relationship adjustment?
5. Do self–other agreement ratings of low- and high-visibility problem traits predict relationship adjustment?
6. Does the level of relationship adjustment moderate the level of self–other agreement of psychological problems?

## Methods

### Sample

The sample consisted of 101 couples (*n* = 202) from three different groups: divorcing couples in family court (*N* = 61, [*n* = 122]) undergoing child custody evaluations, and married organ transplant couples (*N* = 20, [*n* = 40]) where one partner was clinically ill with end-stage liver disease and undergoing liver transplant evaluation. These two groups were convenience samples gathered at a private forensic and clinical practice in Honolulu, Hawaii. Non-clinical married couples (*N* = 20, [*n* = 40]) were independently gathered as a control “non-clinical” group. All the respondents provided written and verbal informed consent, including research participation. No reimbursement or incentives were provided for participation. The study was approved by the Argosy University, Hawai’i, Institutional Review Board, Honolulu, Hawaii.

Couples were sampled to demonstrate a range of dyadic adjustment and marital quality. Divorcing couples were expected to demonstrate higher levels of marital maladjustment (Emery, 1982; Maccoby and M'Nookin, 1992; Johnston, 1994), given that their marriage had failed and they were engaged in a conflicted legal dispute. Transplant couples were used as a contrast group as they were receiving clinical services for a significant life stressor within an intact marriage (Miyazaki et al., 2010). Non-clinical couples served as a control “non-clinical” group for low conflict dyadic adjustment.

The average age of spouses was 42 years old (*M* = 42.25, *SD* = 10.77). All had eighth-grade or higher reading skills based on educational achievements (*M* = 15 years, *SD* = 1.3 years). The spouses represented a variety of ethnicities: Caucasian (41% husbands, 33% wives), African American (28% wives, 27% husbands), Asian-American (14% wives, 10% husbands), Latino (12% wives, 8% husbands), Pacific Islander (3% husbands, 5% wives), and other (12% wives, 11% husbands).

*A priori* power analysis (Faul et al., 2007) suggested that the current sample size was adequate to confidently detect moderate-to-large effects (*n* = 89, *f*^2^ = 0.15; *n* = 46, *f*^2^ = 0.30) on *F* tests in linear multiple regression analyses. Confidence for small effects would require a larger sample size.

### Design and measures

A reciprocal design (Kurtz and Sherker, 2003) using cross-informant assessment (Achenbach and Rescorla, 2003) was employed. The reciprocal assessment examines self–other agreement by applying paired measures (i.e., self and other reports) of ratings for individual and reciprocal comparisons on the same set of Likert items; for example, “I feel satisfied with my spouse or partner.” The Achenbach Adult Forms are self-administered behavior checklists and are among the most widely used and well-validated psychological assessment measures currently in use.

The Achenbach System of Empirically Based Assessment (ASEBA, Achenbach and Rescorla, 2003) adult forms [Adult Self-Report (ASR) for ages 18–59; Adult Behavior Checklist (ABCL) for ages 18–59)] were used to measure self and other behavior ratings from both marital partners. The test–retest reliability of ASEBA adult forms was reported using one-week test–retest *r*s in the 0.80s and 0.90s for most scales, with none <0.71 (Achenbach and Rescorla, 2003). The scales demonstrated good internal consistency, with mean alpha coefficients of 0.83 and 0.85 for the empirically based problem scales. Cross-informant *r*s between ASR and ABCL scores averaged 0.40 for the empirically based problem scales (Achenbach and Rescorla, 2003).

ASEBA is based on two higher-order factors for understanding personality and behavior: externalized and internalized behaviors (Achenbach and Rescorla, 2003). Externalized behavior problems are characterized by clearly visible displays of under-controlled emotions (e.g., belligerence and irritability), rule-breaking behaviors, and difficulties with interpersonal relationships (Achenbach and Edelbrock, 1978; Hinshaw, 1992). Conversely, internalized behavior problems are characterized by less visible displays involving over-controlled emotions and feelings of depressed mood, worthlessness, inferiority, and dependency that may include demands for attention and social withdrawal, which may be difficult to interpret by an observer (Achenbach and Edelbrock, 1978; McCulloch et al., 2000). Internalized and externalized scales were selected for the examination of trait visibility. Internalized standard scores (*M* = 50, *SD* = 10) represented low-visibility problems, while externalized standard scores (*M* = 50, *SD* = 10) represented high-visibility problems.

The Achenbach Total Problems Scale is a parsimonious index of psychological adjustment. It represents a global index of problematic spousal functioning, as appraised by the individual and their partner. Total problems standard scores (*M* = 50, *SD* = 10) represented summative internalized, externalized, and other clinical syndromes that impact relationship functioning (e.g., thought and attention problems). The ASR and ABCL Total Problems Scales demonstrated good to excellent reliability: Cronbach’s alpha of 0.97 in the normative samples (Ns = 295 and 402), with a test–retest *r* of 0.94 and 0.92, respectively (Achenbach and Rescorla, 2003).

The Achenbach scales permit reciprocal comparisons of qualified agreement between informants. Qualifying (*Q*) correlations (Achenbach and Rescorla, 2003) represent the relationship between scores obtained from two sources on a shared set of variables. *Q* correlations range from −1.00, indicating complete disagreement, to +1.00, indicating complete self–other agreement. Scale temporal stability was indicated by *r*s ranging from 0.69 over a two-year interval to 0.58–0.60 over 39–44 months and 0.43–0.53 over 10 years (Achenbach and Rescorla, 2003).

The ASR Total Problems Scale was internally consistent for self-reported problems (Cronbach’s alpha: 0.85 for wives and 0.82 for husbands), and the ABCL Total Problems Scale had good internal consistency (Cronbach’s alpha of 0.90 for husbands and 0.91 for wives) for other reports. The absolute agreement of cross-spousal reports for husband (self) and wife (other) had a standardized internal consistency alpha of 0.85. The average intraclass correlation (ICC) for husband-self-wife-other was 0.85 (95% CI = 0.80–0.89, *p* < 0.001). The absolute agreement of cross-spousal reports for wife (self) and husband (other) yielded a standardized internal consistency alpha of 0.83. The average ICC for wife-self-husband-other ratings was 0.84 (95% CI = 0.78–0.88, *p* < 0.001). Spousal ratings for *Q* yielded a coefficient alpha of 0.88. The weighted average for *Q* correlations showed an absolute agreement ICC of 0.88 (95% CI = 0.85–0.91, *p* < 0.05).

Representing low-visibility psychological problem behaviors, the ASR internalizing scale had acceptable internal consistency for self-reported problems (Cronbach’s alpha of 0.71 for wives and 0.77 for husbands), and the ABCL internalizing scale had acceptable internal consistency (Cronbach’s alpha of 0.73 for husbands and 0.71 for wives) for other-reported low-visibility problems. The absolute agreement of cross-spousal reports of low-visibility problem behaviors for husband (self) and wife (other) ratings had a standardized internal consistency alpha of 0.63. The average ICC for husband-self-wife-other ratings was 0.63 (95% CI = 0.50–0.73, *p* < 0.001). The absolute agreement of cross-spousal reports for low-visibility problem behaviors yielded a standardized internal consistency alpha of 0.66 for wife (self) and husband (other) ratings. The average ICC for wife-self-husband-other ratings was also 0.66 (95% CI = 0.54–0.75, *p* < 0.001).

Representing high-visibility psychological problem behaviors, the ASR externalizing scale had acceptable internal consistency for self-reported problems (Cronbach’s alpha of 0.76 for wives and 0.71 for husbands), and the ABCL externalizing scale had good internal consistency (Cronbach’s alpha of 0.89 for husband and 0.87 for wife) for other reports. Cross-spousal reports for high-visibility psychological problems on husband (self) and wife (other) ratings had a good absolute agreement with a standardized internal consistency alpha of 0.87. The average ICC for husband-self-wife-other ratings was 0.868 (95% CI = 0.82–0.91, *p* < 0.001). Cross-spousal reports for high-visibility psychological problems, based on wife (self) and husband (other) ratings, exhibited an acceptable alpha of 0.65. The average ICC for wife-self-husband-other ratings was 0.65 (95% CI = 0.53–0.75, *p* < 0.001).

The Dyadic Adjustment Scale (DAS; Spanier, 2001) was used to measure relationship adjustment as an indicator of marital quality rated by both spouses. The DAS is a 32-item scale assessing the quality of the relationship between partners. Partners are asked to indicate the approximate extent of agreement or disagreement in 15 areas: handling of family finances, matters of recreation, religious beliefs, demonstrations of affection, friendships, sexual relations, conventionality (proper behavior), life philosophy, ways of dealing with in-laws, shared aims and goals, time spent together, major decision-making, household tasks, leisure time and interests, and career decisions. Couples are asked to indicate how often they engage in behavior in seven areas: confiding in their mate, quarreling with their mate, engaging in activities together, experiencing fatigue affecting their sexual relationship, assessing their overall happiness in the relationship, and expressing their feelings about the future of the relationship (Spanier, 2001).

The DAS is a widely used self-report measure of relationship quality. The DAS was originally formed on a sample of 218 married and 41 divorced Caucasian couples. The DAS has four scales (consensus, satisfaction, affectional expression, and cohesion) that contribute to an overall dyadic adjustment score. The Overall Dyadic Adjustment scale (DASODA) was chosen as the outcome variable because it serves as a composite measure of four components of relationship adjustment required for maintaining marital quality. Internal consistency coefficients for DASODA ranged from 0.80 to 0.90 for wives and from 0.69 to 0.77 for husbands in the normative sample (Spanier, 2001). Temporal stability studies indicate that DAS scores are reasonably stable over relatively long periods (0.78 to 0.98 for 11-week retest; Spanier, 2001).

In the present sample, internal consistency coefficients for DASODA were calculated by combining 64 items for couples (32 items per spouse). Combined couple-mean overall dyadic adjustment scores below 97 indicate relational dysfunction, whereas mean scores above 100 indicate functional relationships (Spanier, 2001). Combined spousal ratings for DASODA yielded a coefficient alpha of 0.70. Spousal DASODA scores showed a weighted average absolute agreement ICC of 0.80 (95% CI = 0.67–0.88, *p* < 0.05).

### Procedures

Instruments were administered in the same order to both marital partners, while seated apart from each other, and were presumed to be well-acquainted couples (Kurtz and Sherker, 2003). Each spouse evaluated themselves and their partner utilizing identical items, resulting in self, other, and dyadic ratings for internalizing, externalizing, and total psychological problems. Each spouse then rated their overall dyadic adjustment. These ratings generated *t*-scores for the corresponding variables of interest, necessitating data transformations to prepare the data for analysis.

### Data transformation

Diagnostic tests assessed the normality of the distributions on the self and other ratings of problems. Some of the variables were positively skewed, and square-root transformation was applied as a remedy (Tabachnick and Fidell, 2007). All the self and other ratings were transformed to equivalent square roots to maintain uniformity. The square-root transformations revealed symmetry for all variables. The square-rooted data were then centered to control multicollinearity by subtracting the grand mean from individual self–other ratings. The transformed scores had a mean of zero (Aiken and West, 1991; Kenny et al., 2006). Self and other difference scores were constructed by subtracting the other rating from the self-rating of the same scale. We calculated self–other differences scores as equally weighted self–other psychological problem ratings (0.50). Pearson correlations for spousal ratings for total psychological problems were 0.51, *p* < 0.05 for other ratings, and insignificant for self-ratings. Interaction variables were constructed by multiplying the centered square root of self and other predictor variables for stepwise, multiple moderator regression analyses.

Reciprocal ratings on matching items also presented issues of data dependence and collineation. Non-independence was controlled by converting variables to reduce multicollinearity by examining combined dyadic ratings instead of the independent partners (Kenny and Ledermann, 2010), and we used family-wise Bonferroni correction. Combined spousal ratings were indicators of couple-overall dyadic adjustment, couple *Q* correlations, and couple self–other problems difference scores to represent the dyad instead of the individual spouse.

Previous investigations (Christensen and Arrington, 1987; Cook and Kenny, 2005) cautioned that summing or averaging scores could lead to unbalanced and biased representations of raters’ levels of cohesion. Therefore, paired-sample *t*-tests were used to explore the similarities or differences between spouse relationship adjustment scores, *Q* correlations, and self–other difference scores before combining the data. Spouse dyadic adjustment did not differ, *t*(101) = 0.57, *p* > 0.05, *d* = 0.06. Spouses *Q* correlation coefficients did not differ, *t*(101) = −1.01, *p* > 0.05, *d* = 0.06. Spouses self–other difference scores did not differ, *t*(101) = 0.00, *p* > 0.05, *d = 0*.00. Wives’ and husbands’ dyadic adjustment was largely similar (*r* = 0.64, *p* < 0.05). Partner *Q* correlations were moderately similar (*r* = 0.34, *p < 0.*05). Self–other difference scores were moderately similar (*r* = 0.46, *p* < 0.05). Similarity within spousal ratings and lack of mean differences justified creating a combined ‘Couple score’ by summing the spouse ratings (Kenny and Ledermann, 2010) to represent dyadic adjustment, self–other agreement, and difference scores for total psychological problems. Tercile splits categorized as low–moderate–high for self–other agreement and overall dyadic adjustment were calculated for research questions 1 and 6.

The data met all assumptions of normality. The data were analyzed using IBM, SPSS Statistics (Version 29). Cohen’s effect sizes (*d*, *f*, 1988) were calculated using public domain calculators (Becker, 1999; Soper, 2014).

## Results

### Demographic variables

Exploratory correlational analyses examined demographic confounds. Spousal age and education demonstrated associations with key variables, and the subsequent analyses treated age and education as covariates.

### Correlations

The dyadic adjustment scores had moderate negative correlations with self–other differences scores (−0.62 for couple TPDI, −0.50 for wife TPDI, −0.51 for husband TPDI, *p* < 0.05). Self-reported total psychological problems were not associated with dyadic adjustment. Other-reported total psychological problems were negatively related to dyadic adjustment (husband/wife: internalizing −0.50/−0.50; externalizing −0.72/−0.61; total problems −0.63/−0.55, *p* < 0.05) and positively associated with self–other differences scores. The husband’s and wife’s other reports for psychological problems were positively correlated (*r* = 0.40, *p < 0*.05). Spousal differences scores were positively related, indicating the reciprocal nature of self–other disagreement.

### Research questions

#### Do different groups of couples differ on the level of relationship adjustment?

Dyadic adjustment differed between the three groups of couples with a large effect, *F* (2, 94) = 23.38, *p < 0.*05, *f*^2^ = 0.81. Non-clinical couples exhibited the highest levels of dyadic adjustment (*M* = 101.45, *SD* = 8.82), transplant couples exhibited intermediate dyadic adjustment (*M* = 86.55, *SD* = 20.66), and divorcing couples exhibited the lowest levels of dyadic adjustment (*M* = 60.08, *SD* = 12.42). Mean differences showed that non-clinical couples exhibited significantly higher dyadic adjustment than divorcing couples, and transplant couples exhibited significantly higher adjustment than divorcing couples.

#### Do different groups of couples differ on self–other agreement ratings for overall psychological problems?

Self–other difference scores differentiated the three couple groups with a large effect, *F* (2, 94) = 22.87, *p* < 0.05, *ɳ*^2^ *partial* = 0.32. Divorcing couples exhibited the highest self–other differences on total psychological problem ratings (*M* = 1.70, *SD* = 2.28), transplant couples exhibited intermediate self–other differences in problem ratings (*M* = −1.92, *SD* = 1.86), and non-clinical couples exhibited the lowest self–other differences for total psychological problems (*M* = −3.25, *SD* = 1.46). Mean differences indicated that divorcing couples exhibited significantly higher self–other total problem difference scores than non-clinical and transplant couples. Non-clinical and transplant couples did not significantly differ in total psychological problem difference ratings.

Self–other qualified correlations significantly differed across the three couple groups with a large effect, *F* (2, 94) = 7.95, *p* < 0.001, *ɳ*^2^ *partial* = 0.14. Non-clinical couples exhibited high-average levels of self–other agreement (*M* = 0.59, *SD* = 0.27), transplant couples had average levels of self–other agreement (*M* = 0.50, *SD* = 0.26), and divorcing couples exhibited low-average levels of self–other agreement (*M* = 0.25, *SD* = 0.24). Mean differences suggested that non-clinical couples exhibited significantly higher self–other agreement than divorcing couples. Transplant couples exhibited higher self–other agreements than divorcing couples. Non-clinical and transplant couples were similar in agreement.

#### Do different groups of couples differ on self–other agreement ratings for low- and high-visibility traits of psychological problems?

Self–other agreement ratings for low-visibility problems (internalizing) differentiated the three couple groups with a large effect, *F* (2, 94) = 11.21, *p* < 0.001, *ɳ*^2^ *partial* = 0.19. Divorcing couples exhibited the highest differences on low-visibility problem agreement (*M* = 1.19, *SD* = 2.05), transplant couples exhibited intermediate differences (*M* = −1.4, *SD* = 2.06), and non-clinical couples exhibited the lowest difference for low-visibility problem agreement (*M* = −2.24, *SD* = 1.26). Mean differences suggest that divorcing couples exhibit significantly higher differences for low-visibility problems than non-clinical couples and transplant couples. Non-clinical and transplant couples were similar in low-visibility problem ratings.

The self–other agreement ratings for high-visibility problems (externalizing) differentiated the three groups with a large effect, *F* (2, 94) = 28.48, *p* < 0.001, *ɳ^2^ partial* = 0.37. Divorcing couples exhibited the highest differences on high-visibility problem agreement (*M* = 1.96, *SD* = 2.28), transplant couples exhibited intermediate differences (*M* = −2.83, *SD* = 2.27), and non-clinical couples exhibited the lowest differences on high-visibility problem agreement (*M* = −3.1, *SD* = 1.34). Mean differences suggest divorcing couples exhibited significantly higher differences for high-visibility problem agreement than non-clinical couples and transplant couples. Non-clinical and transplant couples were similar in high-visibility problem agreement.

#### Do self–other agreement ratings of total psychological problems predict overall dyadic adjustment?

The self–other agreement rating model for total psychological problems is predictive of overall dyadic adjustment with a large effect, *R*^2^ = 0.50, *Adj. R*^2^ = 0.47, *F* (6, 94) = 15.87, *p* < 0.001, *f*^2^ = 1.0, and without interference of multicollinearity (VIF = <1.5). Spouses’ self-rated total psychological problems did not predict dyadic adjustment. Wives’ and husbands’ other ratings of each other’s total psychological problems significantly predicted lower dyadic adjustment. Self–other interactions did not predict dyadic adjustment. 50% of the variance in dyadic adjustment was attributed to spousal other ratings of each other on total psychological problems (see Table 1).

**Table 1:** Self-other agreement model coefficients of total psychological problems.

Total psych. problem ratings	Standard beta	*t*	Sig.	Cohen’s *d*
Wife Self Report	0.040	0.540	0.591	0.10
Husband Self Report	0.078	1.04	0.298	0.20
Husband Other Rating of Wife	−0.472	−5.02	<0.001	−1.00
Wife Other Rating of Husband	−0.338	−3.88	<0.001	−0.77
HSelf x WOther Interaction	−0.004	−0.054	0.957	−0.01
WSelf x HOther Interaction	0.017	0.206	0.837	0.04

#### Do self–other agreement ratings for low- and high-visibility problem traits predict relationship adjustment?

The self–other agreement rating model for low-visibility problems predicted overall dyadic adjustment with a large effect, *R*^2^ = 0.45, *Adj. R*^2^ = 0.41, *F* (6, 94) = 12.91, *p* < 0.001, *f*^2^ = 0.82, without interference of multicollinearity (VIF = <1.5). Husbands’ and wives’ self-rated low-visibility problems did not predict overall dyadic adjustment. In contrast, wives’ and husbands’ other ratings of each other’s low-visibility problems significantly predicted lower dyadic adjustment. The husband-self-wife-other interaction did not moderate-dyadic adjustment, but the wife-self-husband-other interaction moderated overall dyadic adjustment positively (see Table 2).

**Table 2:** Self-other agreement model coefficients for low visibility problems.

Internalized problem ratings	Standard beta	*t*	Sig.	Cohen’s *d*
Wife Self Report	0.09	1.15	0.25	0.23
Husband Self Report	0.135	1.68	0.09	0.33
Husband Other Rating of Wife	−0.363	−4.21	<0.001	−0.84
Wife Other Rating of Husband	−0.372	−4.38	<0.001	−0.87
HSelf x WOther Interaction	0.120	1.51	0.133	0.30
WSelf x HOther Interaction	0.169	2.09	<0.05	0.42

The self–other agreement rating model for high-visibility problems predicted dyadic adjustment with a large effect, *R*^2^ = 0.59, *Adj. R*^2^ = 0.56, *F* (6, 94) = 22.87, *p* < 0.001, *f*^2^ = 1.44 without interference of multicollinearity (VIF = <1.7). Husbands’ and wives’ self-rated high-visibility problems did not predict dyadic adjustment; however, their ratings of each other’s high-visibility problems did predict lower dyadic adjustment. Additionally, neither spousal interactions moderated overall dyadic adjustment (see Table 3).

**Table 3:** Self-other agreement model coefficients of high visibility problems.

Externalized problem ratings	Standard beta	*t*	Sig.	Cohen’s *d*
Wife Self Report	−0.072	−1.06	0.288	−0.212
Husband Self Report	0.013	0.194	0.846	0.038
Husband Other Rating of Wife	−0.568	−6.55	<0.001	−1.31
Wife Other Rating of Husband	−0.276	−3.20	<0.01	−0.064
HSelf x WOther Interaction	−0.042	−0.621	0.536	−0.124
WSelf x HOther Interaction	−0.015	−0.213	0.832	−0.04

#### Does the level of relationship adjustment moderate the level of self–other agreement ratings of psychological problems?

The level of dyadic adjustment had a significant correlation with level of self–other agreement with a moderate effect, *F* (2, 94) = 3.88, *p* < 0.05, *ɳ*^2^ *partial* = 0.08. Couples low in dyadic adjustment (<25%, *N* = 42, [*n* = 84]) exhibited the lowest self–other agreement (*M* = 1.45, *SD* = 0.55). Couples with a moderate level of dyadic adjustment (25–75%, *N* = 42, [84]) exhibited moderate levels of self–other agreement (*M* = 1.95, *SD* = 0.82). Couples with a high level of dyadic adjustment (>75%, *N* = 17, [*n* = 34]) exhibited the highest level of self–other agreement (*M* = 2.05, *SD* = 0.65). The level of self–other agreement significantly differentiated between low and moderate levels of dyadic adjustment (*MD* = −0.40, *SE* = 0.15, *p* < 0.05). There were no significant differences between moderate and elevated levels of dyadic adjustment in the level of self–other agreement (*MD* = 0.01, *SE* = 0.20, *p* > 0.05). Regression analysis revealed the level of dyadic adjustment had a small moderating effect on the level of self–other agreement, *R*^2^ = 0.11, *Adj. R*^2^ = 0.11, *F* (1, 99) = 12.71, *p* < 0.001, *f*^2^ = 0.12 without interference in multicollinearity (VIF = <1.0). The level of dyadic adjustment was positively affected by the level of self–other agreement (*t* = 3.43, *p < 0*.001, *β* = 0.33). The results indicate dyadic adjustment has a significant moderating effect on self and other agreements for psychological problem ratings.

## Discussion

Since the mid-1950s, social and personality psychologists have examined self–other agreement and searched for potential moderators of relationship consensus. Moderators of self–other agreement for personality traits were acquaintanceship (Funder and Colvin, 1988), liking or disliking (McCrae et al., 1998), and relationship quality (Watson et al., 2000b; Kurtz and Sherker, 2003) in all types of relationships. The results of these investigations have been disappointing because none of these factors were found to moderate self–other agreement (Kurtz and Sherker, 2003). Luo and Klohnen (2005) and Gaunt (2006) found self–other agreement and relationship satisfaction have yielded positive findings and are consistent with the results of this present study.

The way spouses see each other directly influences their reported level of marital quality as reflected in dyadic adjustment, and relationship adjustment influences the level of self–other agreement. Our findings replicate studies linking perceived partner similarity and marital satisfaction (Murstein and Beck, 1972; Luo and Klohnen, 2005; Gaunt, 2006). Unlike previous studies, which examined strangers, friends, dating, newlyweds, and married couples, the current study included three marital groups expected to exhibit a high degree of familiarity and a wide range of relationship adjustments.

**Figure 1:**
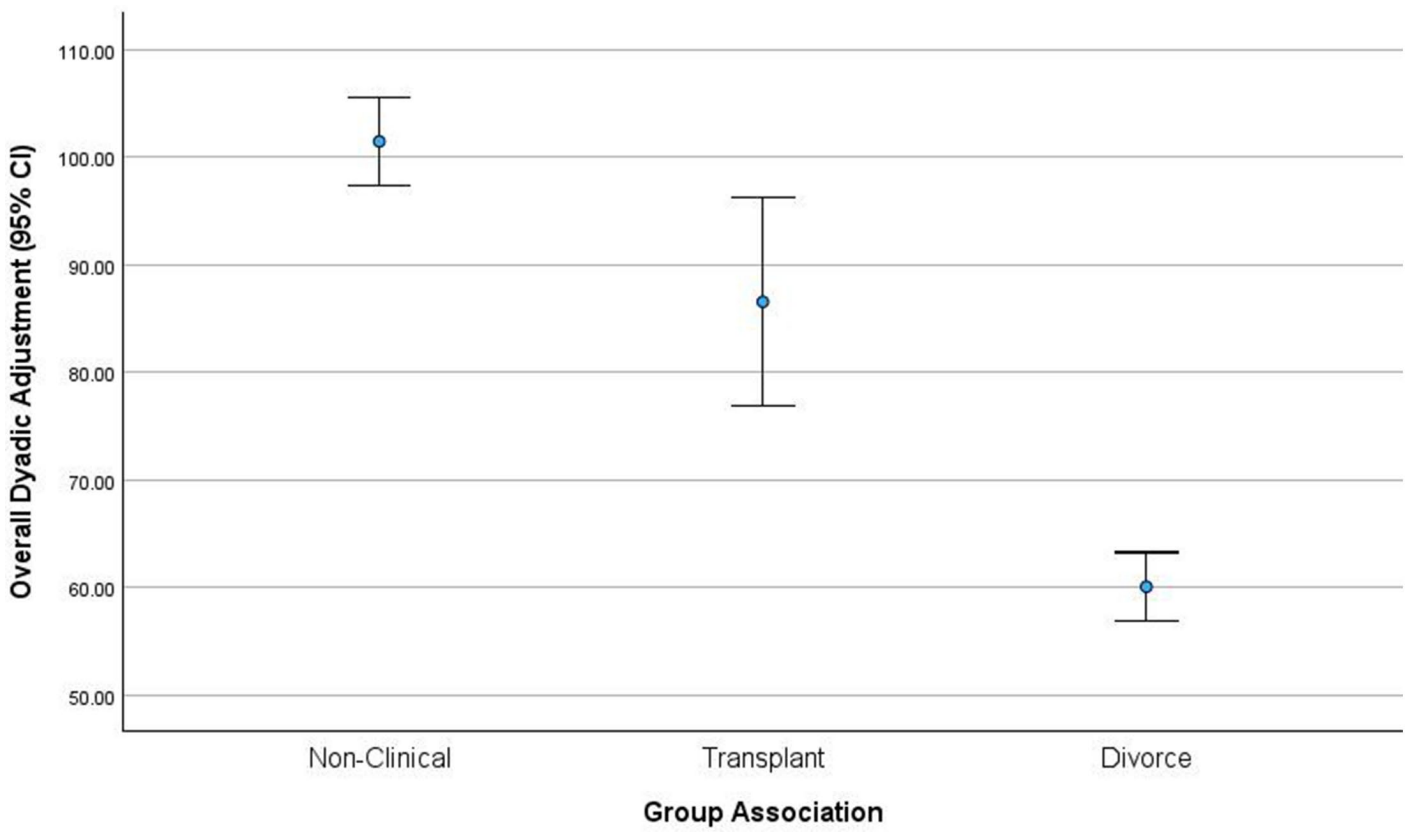
Level of dyadic adjustment.

The assumption that the three couple groups would exhibit a linear range (low–medium–high) of dyadic adjustment was confirmed. As expected, divorcing spouses exhibited extreme levels of self–other disagreement and lower levels of dyadic adjustment, while non-clinical couples exhibited the lowest differences in total psychological problem recognition, along with the highest levels of self–other agreement and dyadic adjustment. 90% of transplant couples exhibited moderate-to-high dyadic adjustment, and 100% of non-clinical couples had moderate-to-high dyadic adjustment versus 100% of divorcing couples who had low-to-moderate dyadic adjustment (see Figure 1).

**Figure 2:**
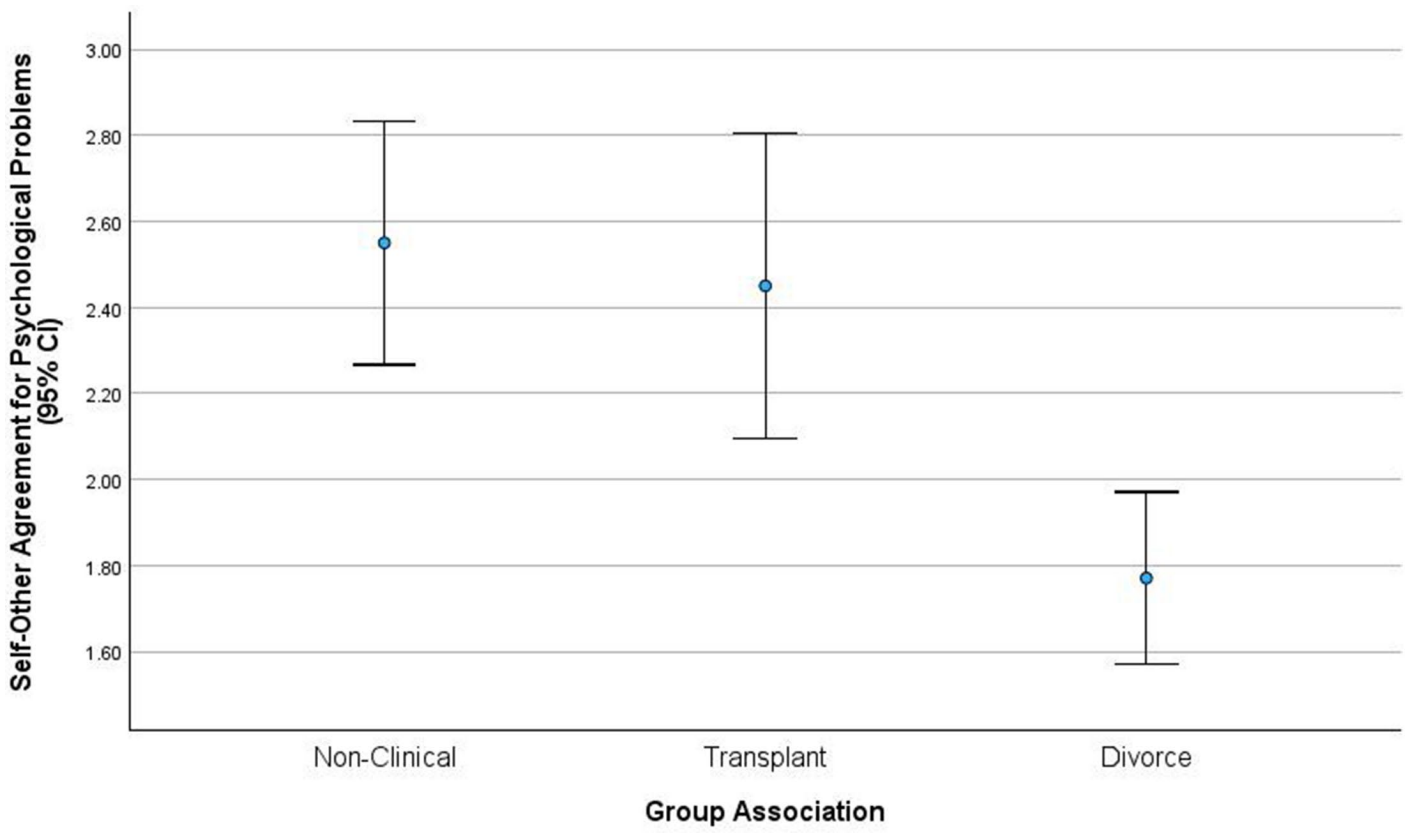
Self-other agreement model.

The assumption that the three couple groups would demonstrate a linear range (low–medium–high) of self–other agreement overlapped; the results suggested that self–other agreement may have a curvilinear relationship to relationship quality (see Figure 2). In other words, self–other agreement and dyadic adjustment rise together until a point where disagreement ultimately shatters the perceptions of marital quality and diminishes the couple’s ability to adjust. For the most part, the transplant group had similar levels of dyadic adjustment to non-clinical couples, although there appeared to be some heterogeneity across the three groups for self–other agreement and dyadic adjustment. We could postulate ‘maladjusted-non-clinical’ couples and ‘well-adjusted-divorcing couples’, although none of the non-clinical couples demonstrated low degrees of dyadic adjustment and none of the divorcing couples demonstrated high degrees of dyadic adjustment.

**Figure 3:**
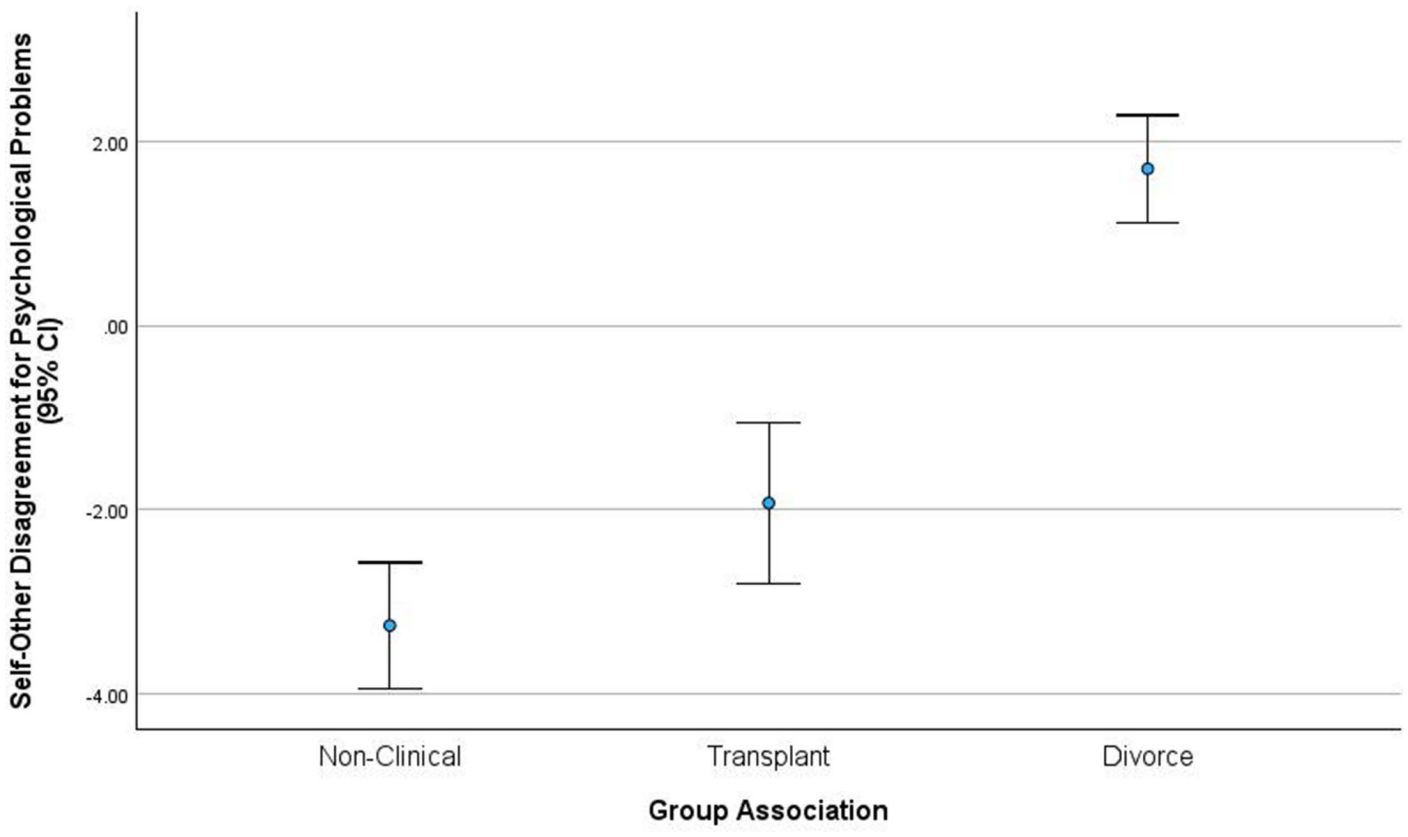
Self-other disagreement model.

The self–other agreement indicator correlations suggest that our findings are consistent with previous studies as non-clinical couples demonstrate moderately elevated levels of self and other agreements (exhibited in high self–other correlations and minimal ASR-ABCL difference effect sizes). Custody spouses demonstrated extreme levels of self–other disagreement (see Figure 3), reflected by large effect sizes, with other ratings contributing to the largest effects. In divorcing couples, harsh other ratings (John and Robins, 1994) appear to be a potent underpinning of problematic attributions that contribute to poor reciprocal interactions. This indicator can be used to delineate distraught and non-distraught couples (Bradbury and Fincham, 1990).

We found that relationship quality demonstrated moderate-to-large effect sizes as a moderator of self–other agreement ratings for psychological problems. Multiple moderator regression analyses disaggregated complex interaction variables reflecting the highly negative way that clinical husbands and wives view each other. In many respects, our findings are consistent with the conclusion reached by Watson et al. (2000b). As the level of dyadic satisfaction moderates spousal ratings of each other, the spouses relied upon their perceptions of marital quality as a foundation for judgmental attributions about each other’s personality qualities. Non-clinical relationships appear satisfied and fortified with a shared well-regard, cooperation, and emotional stability, which is essentially adaptive dyadic adjustment. Divorcing relationships appear dissatisfied and weakened with uncooperative, unreliable, and emotionally moody behaviors, which are apparent signs of maladaptive adjustment.

A meta-analysis of 50,000 relationship satisfaction measures discovered no differences between men and women, suggesting that both genders are barometers of relationship satisfaction (Johnson et al., 2022). Our tests of higher-order interaction effects for mutual influence (non-independence) of inter-spousal appraisals provided partial support for the mutual influence model with primacy given to the interaction between the wife’s view of themselves and the husband’s view of their wives. These findings are consistent with the view that wives’ perceptions are more determinative of dyadic adjustment and overall quality (Luo and Klohnen, 2005). Wives appear to be better attuned to dyadic pressures, modulating relational attitudes, consensus, and contexts, making them a barometer for reflecting the quality of the relationship.

Historically, marital interactions had stronger implications for women than men regarding relationship satisfaction (Luo and Klohnen, 2005). Marital satisfaction is moderated by gender, with smaller effects for fathers and larger for mothers (Ronaghan et al., 2023). Other moderators included the mother’s age, the father’s educational level, the length of the relationship, and the number of children. The largest effects suggested that marital satisfaction were moderated by older mothers, fathers with lower education, longer relationship length, and a greater number of children (Ronaghan et al., 2023).

Adaptive dyadic adjustment in married couples is contingent upon natural drives to develop and maintain close relationships that revolve around supportive and caring behaviors (Baumeister et al., 2001). Our results indicate marital quality improves when husbands are attuned to their wives’ expressions of internalized traits. In other words, husbands who can adaptively adjust to their spouses’ displays of emotional withdrawal, low self-esteem, and demands for attention will have increased relationship quality. Positive verbal and non-verbal forms of relationship communication are more satisfying, whereas negative behaviors and communication patterns are strongly associated with relationship dissatisfaction (Gottman, 1994). Spouses who focus on negative experiences over positive experiences (Baumeister et al., 2001) need to adjust with efforts toward decreasing negative interactions as opposed to increasing positive interactions (Gottman, 1994).

In the transplant marital group, transplant wives were typically the designated caregivers for their recipient husbands. Their participation was a requirement for the transplant evaluation. Transplant candidates and caregivers may present with socially desirable responses as a coping strategy in the transplant evaluation, such as in a job selection situation (Rothstein and Goffin, 2006). Transplant couples are typically highly anxious to be listed and obtain a liver transplant since the likely alternative is the realistic threat of declining health and death. This stressor on an otherwise intact marriage may have triggered socially desirable response patterns that minimized their sense of caregiver distress as they were fearful of it affecting the potential transplant listing status (Goetzinger et al., 2012). Nevertheless, the transplant group demonstrated a moderate range of self–other agreement, differences, and relationship satisfaction between non-clinical and custody-disputing couples.

In the divorcing group, situational demand characteristics of the family court psychological evaluation may have activated self-enhancement (Vernooij, 2023) and other-devaluing biases (Paulhus, 1984; John and Robins, 1994). Divorcing women tend to be angrier, express less marital satisfaction, are more likely to initiate the divorce, and are more favorable to divorce than their husbands (Kelly, 1991). The highly polarized other ratings appear to be motivated perceptions, which reflect the divorcing couple’s loss of a commonly shared reality, a crucial factor in understanding interparental conflict. Furthermore, threats inherent in the custody dispute, including the potential loss of access to children and shame and anger at the marital break-up, may trigger defensive blaming that underlies self-favoring biases (Ehrenberg et al., 1996). These distortions impact the empathy of a person’s perspective and accuracy in predicting each other’s behavior (Kruglanski, 1989), including the evaluation of parenting capabilities. In these instances, motivations in disputing marital partners may have their perspectives pulled in opposite directions, offering a justification for hostility toward the other spouse (Kruglanski, 1989).

The present findings indicate a restricted range in all the groups for self-ratings, raising the possibility of self-enhancing biases in the transplant and custody groups as their self-reported problems did not differ from the non-clinical couple group. With respect to self-enhancing bias in divorcing couples that are custody disputing, a small number of research studies discuss the tendency of custody litigants to “fake good” in psychological evaluations (Singer et al., 2008; Archer et al., 2012). Child custody disputes are extremely stressful and threaten attachment security in both adults and children and may be singular in their potential for activating both self-enhancing and other-devaluing biases. Self-enhancing and other-devaluing biases may be reciprocal responses in the custody-disputing group, reflecting an accusation-blame cycle, which characterizes distressed couples. Other harshness ratings appear to be extreme fundamental attribution errors, reflecting observers’ readiness to draw harsh dispositional conclusions about their partner. This often occurs when the observer cannot or will not moderate their inferences by adopting the actor’s perspective or imagining themselves in the other’s shoes (Ehrenberg et al., 1996; Savitsky et al., 2001). This lack of empathy is a central dynamic in high-conflict divorce and custody disputes and a threat to the wellbeing of children (Amato, 1991, 1993, 2000).

In contrast to the studies of Watson et al. (2000a, 2000b), the present results show highly visible behaviors and low-visibility behaviors both significantly reflecting an inner disposition of negativity in the distressed couple groups. Relationship quality has a positive impact on the overall adjustment of the couple, most specifically when husbands are empathic to their wives’ low-visibility processes. This moderator influences agreement and relationship satisfaction, which can be utilized in practice to reduce conflictual patterns that couples endure. Non-clinical couples exhibited the lowest levels of self–other differences and the highest level of dyadic adjustment, whereas the opposite was true for custody litigants. Transplant couples showed a broad range of agreement and marital adjustment.

If consensus in self and other agreements is a criterion for accuracy in personality judgments (Funder, 1995), a poor relationship detracts from accuracy in self and other appraisals. Relationship appraisals are driven by memories, interpretations, and attitudes influenced by past experiences and expectations about the future of the relationship (Reis et al., 2002). The non-clinical group demonstrated minimal mean differences and patterns of correlations in self- and other appraisals, suggesting an elevated level of relationship consensus; non-clinical couples also demonstrated higher degrees of profile similarity in contrast to the other groups. As affirmed by the lengthy tradition of social and personality research, self–other agreement and self–other differences both appear as measures of appraisal accuracy and dyadic adjustment.

### Practical applications

This applied clinical assessment study utilized commonly available assessment tools. Cross-informant assessment methods are particularly useful in psychological evaluations, where a relationship system is being examined, for example, in marriage, divorce, and family assessments, or in other situations, where self-informant correspondences or differences may have clinical significance. The findings have applications to treatment planning and implementing dyadic or family interventions, or evaluation for pre-marital or post-divorce adjustment. Divorce education models focus on shifting parents’ blaming toward concern about the children’s wellbeing. Self–other agreements, difference scores, and *Q* correlations have the potential as clinical and research indices of interparental conflict (IPC), serving as measures of potential acrimony (Emery, 1982; Sbarra and Emery, 2005, 2008). IPC is noted as the most toxic factor in a child’s experience of divorce (Amato, 1991, 1993, 2000). Parents could develop stronger relationships with their children with strategies and methods meant to develop and display warm/receptive attitudes, delineate clear boundaries and consequences, and develop efficacy in helping their child identify emotions and express them to manage conflicts.^1^

### Limitations and research recommendations

The findings highlight the situational aspects of data sampling and serve as a reminder to applied assessment clinicians of the potential for biases when situational demand characteristics may be present. Convenience samples are easily criticized for potential bias; however, this sample was the most cost-effective and met the time frames required for the original dissertation study. Self-enhancement in self-report forms also remains easily criticized and difficult to address (Schwarz, 1999). Current attempts to assess positive impression management have revealed that individuals with personality dysfunction tend to suppress more than 50% of problematic traits (Williams et al., 2019). Other findings suggest that discrepant self and other ratings in married couples were not linked to indicators of response distortions (Muller and Moshagen, 2019). Nevertheless, self-report measures of personality and co-parenting, linked with other reports from familiar informants, remain valid, reliable, and predictive regardless of impression management efforts (Feinberg et al., 2012; Kim et al., 2019).

Further research on self and other agreements with heterogeneous, clinical, and non-clinical couples’ contrasting alternative methods of quantifying self–other agreements would strengthen the model. Future research on divorcing couples who are not involved in child custody disputes is necessary. Not all divorcing couples are acrimonious and litigious. A self–other agreement study of non-litigating, divorcing couples would contrast these two groups of divorcing parents, although some researchers have noted few differences in the level of anger and disparagement between litigating and non-litigating couples (Kelly, 1991). The degree to which intensification of interparental conflict is the result of the divorce process, including litigation and the custody evaluation process itself, or pre-existing personality or relationship variables, remains unclear.

Although the reciprocal design utilized in this study is promising, larger sample sizes would address the attenuation of power for interaction variables (McClelland and Judd, 1993). In this study, the length of the relationship was not examined. This variable may illuminate whether developmental factors and length of relationship moderate self–other agreement.

## Data Availability

The raw data supporting the conclusions of this article will be made available by the authors, without undue reservation.

## Footnotes

^1^ https://www.child-encyclopedia.com/divorce-and-separation/according-experts/how-parents-can-help-children-cope-separationdivorce
