# Supplementary material for: Assessing self–other agreement and dyadic adjustment in marital dyads
Source: Front Psychol. 2024 Nov 15;15:1363165. doi: 10.3389/fpsyg.2024.1363165 (PMC11604461; doi:10.3389/fpsyg.2024.1363165)
Supplement: Supplementary file 1 [file Data_Sheet_1.PDF]

Univariate Analysis of Variance

Notes

Output Created		20-DEC-2023 18:40:30
Comments		
Input	Data	C: \Users\jdwir\OneDrive\Marv in Research\DATA Sets\DyadicData_Whole Lot_122 (2023).sav
	Active Dataset	DataSet1
	Filter	<none>
	Weight	<none>
	Split File	<none>
	N of Rows in Working Data File	101
Missing Value Handling	Definition of Missing	User-defined missing values are treated as missing.
	Cases Used	Statistics are based on all cases with valid data for all variables in the model.

Notes

Syntax		UNIANOVA Level_CplQscore BY Level_CplDASODA WITH YearsEd Age YearsEdWif AgeWif<br><br>/RANDOM=Level_CplDASODA<br>/METHOD=SSTYPE(3)<br>/INTERCEPT=INCLUDE<br>/PLOT=PROFILE (Level_CplDASODA) TYPE=LINE ERRORBAR=CI MEANREFERENCE=NO YAXIS=AUTO<br>/EMMEANS=TABLES (Level_CplDASODA) WITH (YearsEd=MEAN Age=MEAN YearsEdWif=MEAN AgeWif=MEAN) COMPARE<br>ADJ(BONFERRONI)<br>/PRINT ETASQ DESCRIPTIVE HOMOGENEITY OPOWER<br>/CRITERIA=ALPHA(.05)<br>/DESIGN=YearsEd Age ...
Resources	Processor Time	00:00:00.14
	Elapsed Time	00:00:00.13

Between-Subjects Factors

		Value Label	N
Level_CplDASODA	1.00	Blw-Avg Couple Adjustment	42
	2.00	Avg Couple Adjsutment	42
	3.00	Above-Avg Couple Adjustment	17

Descriptive Statistics

Dependent Variable: Level_CplQscore

Level_CplDASODA	Mean	Std. Deviation	N
Blw-Avg Couple Adjustment	1.4524	.55005	42
Avg Couple Adjsutment	1.9524	.82499	42
Above-Avg Couple Adjustment	2.0588	.65865	17
Total	1.7624	.73687	101

Levene's Test of Equality of Error Variances[a]

Dependent Variable: Level_CplQscore

F	df1	df2	Sig.
1.857	2	98	.162

Tests the null hypothesis that the error variance of the dependent variable is equal across groups.

a. Design: Intercept + YearsEd + Age + YearsEdWif + AgeWif + ...

Tests of Between-Subjects Effects

Dependent Variable: Level_CplQscore

Source		Type III Sum of Squares	df	Mean Square	F	Sig.
Intercept	Hypothesis	5.949	1	5.949	12.877	<.001
	Error	42.413	91.809	.462[a]		
YearsEd	Hypothesis	1.696	1	1.696	3.816	.054
	Error	41.769	94	.444[b]		
Age	Hypothesis	.118	1	.118	.267	.607
	Error	41.769	94	.444[b]		
YearsEdWif	Hypothesis	4.158	1	4.158	9.356	.003
	Error	41.769	94	.444[b]		
AgeWif	Hypothesis	.239	1	.239	.539	.465
	Error	41.769	94	.444[b]		
Level_CplDASODA	Hypothesis	3.454	2	1.727	3.886	.024
	Error	41.769	94	.444[b]		

Tests of Between-Subjects Effects

Dependent Variable: Level_CplQscore

Source		Partial Eta Squared	Noncent. Parameter	Observed Power[c]
Intercept	Hypothesis	.123	12.877	.944
	Error			
YearsEd	Hypothesis	.039	3.816	.489
	Error			
Age	Hypothesis	.003	.267	.080
	Error			
YearsEdWif	Hypothesis	.091	9.356	.857
	Error			
AgeWif	Hypothesis	.006	.539	.112
	Error			
Level_CplDASODA	Hypothesis	.076	7.772	.689
	Error			

a. .014 MS(Level_CplDASODA) + .986 MS(Error)

b. MS(Error)

c. Computed using alpha = .05

Expected Mean Squares[a,b]

Source	Variance Component		
	Var (Level_CplDASODA)	Var(Error)	Quadratic Term
Intercept	.388	1.000	Intercept
YearsEd	.000	1.000	YearsEd
Age	.000	1.000	Age
YearsEdWif	.000	1.000	YearsEdWif
AgeWif	.000	1.000	AgeWif
Level_CplDASODA	28.227	1.000	
Error	.000	1.000	

a. For each source, the expected mean square equals the sum of the coefficients in the cells times the variance components, plus a quadratic term involving effects in the Quadratic Term ...

b. Expected Mean Squares are based on the Type III Sums of Squares.

Estimated Marginal Means

Level_CplDASODA

Estimates

Dependent Variable: Level_CplQscore

Level_CplDASODA	Mean	Std. Error	95% Confidence Interval Lower Bound	95% Confidence Interval Upper Bound
Blw-Avg Couple Adjustment	1.528[a]	.107	1.315	1.741
Avg Couple Adjsutment	1.926[a]	.105	1.719	2.134
Above-Avg Couple Adjustment	1.936[a]	.175	1.589	2.284

a. Covariates appearing in the model are evaluated at the following values: HYears of Education = 15.1188, HAge of Participant = 42.6535, WYears of Education = 11.5248, WAge of Participant = 41.2574.

Pairwise Comparisons

Dependent Variable: Level_CplQscore

(I) Level_CplDASODA	(J) Level_CplDASODA	Mean Difference (I-J)	Std. Error	Sig.[b]
Blw-Avg Couple Adjustment	Avg Couple Adjsutment	-.398*	.152	.030
	Above-Avg Couple Adjustment	-.408	.213	.175
Avg Couple Adjsutment	Blw-Avg Couple Adjustment	.398*	.152	.030
	Above-Avg Couple Adjustment	-.010	.205	1.000
Above-Avg Couple Adjustment	Blw-Avg Couple Adjustment	.408	.213	.175
	Avg Couple Adjsutment	.010	.205	1.000

Pairwise Comparisons

Dependent Variable: Level_CplQscore

(I) Level_CplDASODA	(J) Level_CplDASODA	95% Confidence Interval for Difference[b] Lower Bound	Upper Bound
Blw-Avg Couple Adjustment	Avg Couple Adjsutment	-.767	-.029
	Above-Avg Couple Adjustment	-.927	.111
Avg Couple Adjsutment	Blw-Avg Couple Adjustment	.029	.767
	Above-Avg Couple Adjustment	-.510	.490
Above-Avg Couple Adjustment	Blw-Avg Couple Adjustment	-.111	.927
	Avg Couple Adjsutment	-.490	.510

Based on estimated marginal means

*. The mean difference is significant at the .05 level.

b. Adjustment for multiple comparisons: Bonferroni.

Univariate Tests

Dependent Variable: Level_CplQscore

	Sum of Squares	df	Mean Square	F	Sig.	Partial Eta Squared
Contrast	3.454	2	1.727	3.886	.024	.076
Error	41.769	94	.444			

Univariate Tests

Dependent Variable: Level_CplQscore

	Noncent. Parameter	Observed Power[a]
Contrast	7.772	.689
Error		

The F tests the effect of Level_CplDASODA. This test is based on the linearly independent pairwise comparisons among the estimated marginal means.

a. Computed using alpha = .05

Profile Plots

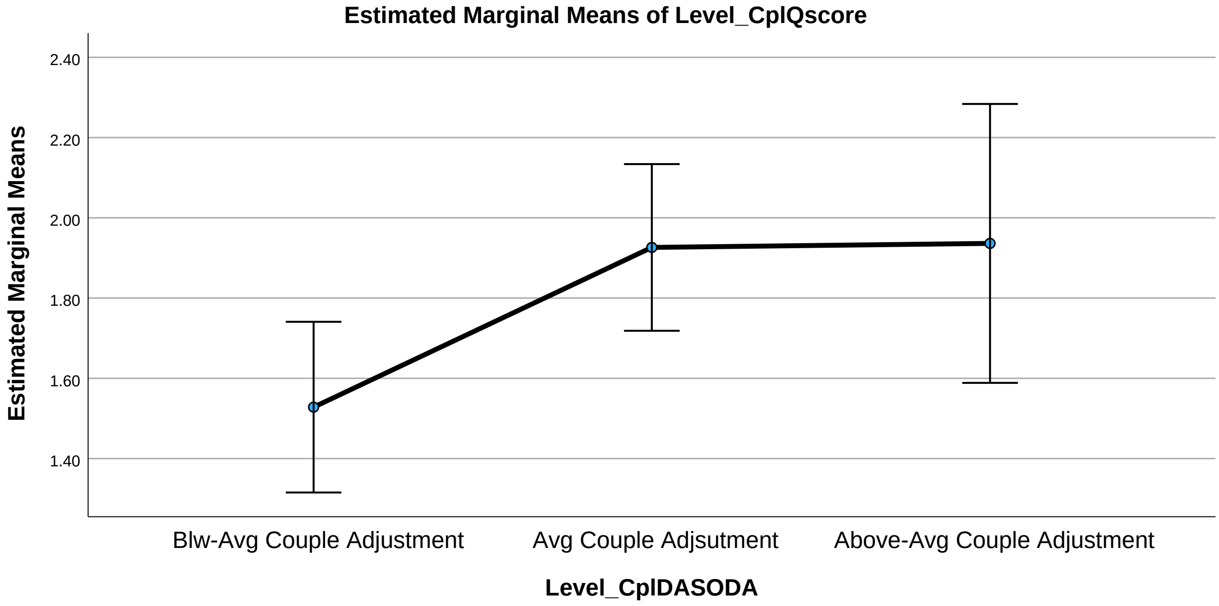

Covariates appearing in the model are evaluated at the following values: HYears of Education = 15.1188, HAge of Participant = 42.6535, WYears of Education = 11.5248, WAge of Participant = 41.2574

Error bars: 95% CI
