# Supplementary material for: Assessing self–other agreement and dyadic adjustment in marital dyads
Source: Front Psychol. 2024 Nov 15;15:1363165. doi: 10.3389/fpsyg.2024.1363165 (PMC11604461; doi:10.3389/fpsyg.2024.1363165)

Regression

Notes

Output Created		20-DEC-2023 16:40:46
Comments		
Input	Data	C: \Users\jdwir\OneDrive\Marvin Research\DATA Sets\DyadicData_Whole Lot_122 (2023).sav
	Active Dataset	DataSet1
	Filter	<none>
	Weight	<none>
	Split File	<none>
	N of Rows in Working Data File	101
Missing Value Handling	Definition of Missing	User-defined missing values are treated as missing.
	Cases Used	Statistics are based on cases with no missing values for any variable used.
Syntax		REGRESSION /DESCRIPTIVES MEAN STDDEV CORR SIG N /MISSING LISTWISE /STATISTICS COEFF OUTS R ANOVA COLLIN TOL CHANGE /CRITERIA=PIN(.05) POUT(.10) TOLERANCE(.0001) /NOORIGIN /DEPENDENT CplDASODA /METHOD=ENTER CntrSqrtWASRInternal CntrSqrtASRInternal /METHOD=ENTER CntrSqrtWABCLInternal CntrSqrtHABCLInternal /METHOD=ENTER HsxWoInternalize WsxHoInternalize /RESIDUALS NORMPROB(ZRESID).
Resources	Processor Time	00:00:00.30
	Elapsed Time	00:00:00.16

Notes

	Memory Required	22848 bytes
	Additional Memory Required for Residual Plots	216 bytes

Descriptive Statistics

	Mean	Std. Deviation	N
CplDASODA	73.5149	22.10639	101
CntrSqrtWASRInternal	.0000	.71068	101
CntrSqrtASRInternal	.0000	.83402	101
CntrSqrtWABCLInternal	.0000	.85776	101
CntrSqrtHABCLInternal	.0000	.81475	101
HsxWoInternalize	.1061	.71778	101
WsxHoInternalize	.1414	.62877	101

Correlations

		CplDASODA	CntrSqrtWASRInternal	CntrSqrtASRInternal
Pearson Correlation	CplDASODA	1.000	-.106	.043
	CntrSqrtWASRInternal	-.106	1.000	.123
	CntrSqrtASRInternal	.043	.123	1.000
	CntrSqrtWABCLInternal	-.519	.223	.150
	CntrSqrtHABCLInternal	-.509	.247	.193
	HsxWoInternalize	.199	-.114	.152
	WsxHoInternalize	.318	-.175	.024
Sig. (1-tailed)	CplDASODA	.	.145	.333
	CntrSqrtWASRInternal	.145	.	.109
	CntrSqrtASRInternal	.333	.109	.
	CntrSqrtWABCLInternal	.000	.012	.067
	CntrSqrtHABCLInternal	.000	.006	.027
	HsxWoInternalize	.023	.127	.065
	WsxHoInternalize	.001	.040	.406
N	CplDASODA	101	101	101
	CntrSqrtWASRInternal	101	101	101
	CntrSqrtASRInternal	101	101	101
	CntrSqrtWABCLInternal	101	101	101
	CntrSqrtHABCLInternal	101	101	101
	HsxWoInternalize	101	101	101
	WsxHoInternalize	101	101	101

Correlations

		CntrSqrtWABCLInternal	CntrSqrtHABCLInternal	HsxWoInternalize
Pearson Correlation	CplDASODA	-.519	-.509	.199
	CntrSqrtWASRInternal	.223	.247	-.114
	CntrSqrtASRInternal	.150	.193	.152
	CntrSqrtWABCLInternal	1.000	.382	-.092
	CntrSqrtHABCLInternal	.382	1.000	-.115
	HsxWoInternalize	-.092	-.115	1.000
	WsxHoInternalize	-.227	-.228	-.042
Sig. (1-tailed)	CplDASODA	<.001	<.001	.023
	CntrSqrtWASRInternal	.012	.006	.127
	CntrSqrtASRInternal	.067	.027	.065
	CntrSqrtWABCLInternal	.	.000	.179
	CntrSqrtHABCLInternal	.000	.	.126
	HsxWoInternalize	.179	.126	.
	WsxHoInternalize	.011	.011	.339
N	CplDASODA	101	101	101
	CntrSqrtWASRInternal	101	101	101
	CntrSqrtASRInternal	101	101	101
	CntrSqrtWABCLInternal	101	101	101
	CntrSqrtHABCLInternal	101	101	101
	HsxWoInternalize	101	101	101
	WsxHoInternalize	101	101	101

Correlations

		WsxHoInternalize
Pearson Correlation	CplDASODA	.318
	CntrSqrtWASRInternal	-.175
	CntrSqrtASRInternal	.024
	CntrSqrtWABCLInternal	-.227
	CntrSqrtHABCLInternal	-.228
	HsxWoInternalize	-.042
	WsxHoInternalize	1.000
Sig. (1-tailed)	CplDASODA	<.001
	CntrSqrtWASRInternal	.040
	CntrSqrtASRInternal	.406
	CntrSqrtWABCLInternal	.011
	CntrSqrtHABCLInternal	.011
	HsxWoInternalize	.339
	WsxHoInternalize	.
N	CplDASODA	101
	CntrSqrtWASRInternal	101
	CntrSqrtASRInternal	101
	CntrSqrtWABCLInternal	101
	CntrSqrtHABCLInternal	101
	HsxWoInternalize	101
	WsxHoInternalize	101

Variables Entered/Removed[a]

Model	Variables Entered	Variables Removed	Method
1	CntrSqrtASRInternal, CntrSqrtWASRInternal[b]	.	Enter
2	CntrSqrtWABCLInternal, CntrSqrtHABCLInternal[b]	.	Enter
3	HsxWoInternalize, WsxHoInternalize[b]	.	Enter

a. Dependent Variable: CplDASODA

b. All requested variables entered.

Model Summary[d]

Model	R	R Square	Adjusted R Square	Std. Error of the Estimate	Change Statistics: R Square Change	F Change	df1	df2	Sig. F Change
1	.120[a]	.015	-.006	22.16815	.015	.722	2	98	.488
2	.645[b]	.416	.392	17.23715	.402	33.045	2	96	<.001
3	.672[c]	.452	.417	16.88214	.035	3.040	2	94	.053

a. Predictors: (Constant), CntrSqrtASRInternal, CntrSqrtWASRInternal

b. Predictors: (Constant), CntrSqrtASRInternal, CntrSqrtWASRInternal, CntrSqrtWABCLInternal, CntrSqrtHABCLInternal

c. Predictors: (Constant), CntrSqrtASRInternal, CntrSqrtWASRInternal, CntrSqrtWABCLInternal, CntrSqrtHABCLInternal, HsxWoInternalize, WsxHoInternalize

d. Dependent Variable: CplDASODA

ANOVA[a]

Model		Sum of Squares	df	Mean Square	F	Sig.
1	Regression	709.410	2	354.705	.722	.488[b]
	Residual	48159.818	98	491.427		
	Total	48869.228	100			
2	Regression	20345.785	4	5086.446	17.119	<.001[c]
	Residual	28523.442	96	297.119		
	Total	48869.228	100			
3	Regression	22078.591	6	3679.765	12.911	<.001[d]
	Residual	26790.637	94	285.007		
	Total	48869.228	100			

a. Dependent Variable: CplDASODA

b. Predictors: (Constant), CntrSqrtASRInternal, CntrSqrtWASRInternal

c. Predictors: (Constant), CntrSqrtASRInternal, CntrSqrtWASRInternal, CntrSqrtWABCLInternal, CntrSqrtHABCLInternal

d. Predictors: (Constant), CntrSqrtASRInternal, CntrSqrtWASRInternal, CntrSqrtWABCLInternal, CntrSqrtHABCLInternal, HsxWoInternalize, WsxHoInternalize

Coefficients[a]

Model		Unstandardized Coefficients B	Unstandardized Coefficients Std. Error	Standardized Coefficients Beta	t	Sig.
1	(Constant)	73.515	2.206		33.328	<.001
	CntrSqrtWASRInternal	-3.522	3.143	-.113	-1.121	.265
	CntrSqrtASRInternal	1.523	2.678	.057	.569	.571
2	(Constant)	73.515	1.715		42.862	<.001
	CntrSqrtWASRInternal	1.936	2.535	.062	.764	.447
	CntrSqrtASRInternal	4.615	2.119	.174	2.178	.032
	CntrSqrtWABCLInternal	-10.449	2.204	-.405	-4.742	<.001
	CntrSqrtHABCLInternal	-10.925	2.348	-.403	-4.652	<.001
3	(Constant)	72.281	1.753		41.235	<.001
	CntrSqrtWASRInternal	2.902	2.514	.093	1.154	.251
	CntrSqrtASRInternal	3.588	2.128	.135	1.686	.095
	CntrSqrtWABCLInternal	-9.591	2.187	-.372	-4.385	<.001
	CntrSqrtHABCLInternal	-9.853	2.341	-.363	-4.209	<.001
	HsxWoInternalize	3.696	2.438	.120	1.516	.133
	WsxHoInternalize	5.955	2.837	.169	2.099	.038

Coefficients[a]

Model		Collinearity Statistics Tolerance	Collinearity Statistics VIF
1	(Constant)		
	CntrSqrtWASRInternal	.985	1.015
	CntrSqrtASRInternal	.985	1.015
2	(Constant)		
	CntrSqrtWASRInternal	.915	1.093
	CntrSqrtASRInternal	.952	1.051
	CntrSqrtWABCLInternal	.832	1.202
	CntrSqrtHABCLInternal	.812	1.232
3	(Constant)		
	CntrSqrtWASRInternal	.893	1.120
	CntrSqrtASRInternal	.905	1.105
	CntrSqrtWABCLInternal	.810	1.235
	CntrSqrtHABCLInternal	.784	1.276
	HsxWoInternalize	.930	1.075
	WsxHoInternalize	.896	1.116

a. Dependent Variable: CplDASODA

Excluded Variables[a]

Model		Beta In	t	Sig.	Partial Correlation	Collinearity .
						Tolerance
1	CntrSqrtWABCLInternal	-.538[b]	-6.054	<.001	-.524	.935
	CntrSqrtHABCLInternal	-.539[b]	-5.975	<.001	-.519	.912
	HsxWoInternalize	.185[b]	1.826	.071	.182	.959
	WsxHoInternalize	.307[b]	3.147	.002	.304	.967
2	HsxWoInternalize	.102[c]	1.271	.207	.129	.942
	WsxHoInternalize	.156[c]	1.932	.056	.194	.907

Excluded Variables[a]

Model		Collinearity Statistics	
		VIF	Minimum Tolerance
1	CntrSqrtWABCLInternal	1.070	.935
	CntrSqrtHABCLInternal	1.096	.912
	HsxWoInternalize	1.043	.957
	WsxHoInternalize	1.034	.953
2	HsxWoInternalize	1.062	.804
	WsxHoInternalize	1.103	.794

a. Dependent Variable: CplDASODA

b. Predictors in the Model: (Constant), CntrSqrtASRInternal, CntrSqrtWASRInternal

c. Predictors in the Model: (Constant), CntrSqrtASRInternal, CntrSqrtWASRInternal, CntrSqrtWABCLInternal, CntrSqrtHABCLInternal

Collinearity Diagnostics[a]

Model	Dimension	Eigenvalue	Condition Index	Variance Proportions (Constant)	CntrSqrtWASRInternal	CntrSqrtASRInternal
1	1	1.123	1.000	.00	.44	.44
	2	1.000	1.060	1.00	.00	.00
	3	.877	1.132	.00	.56	.56
2	1	1.683	1.000	.00	.12	.08
	2	1.000	1.297	1.00	.00	.00
	3	.896	1.370	.00	.09	.89
	4	.807	1.444	.00	.79	.02
	5	.614	1.656	.00	.01	.01
3	1	1.845	1.000	.01	.09	.03
	2	1.249	1.215	.24	.00	.21
	3	1.037	1.334	.22	.04	.07
	4	.893	1.438	.24	.00	.41
	5	.805	1.514	.00	.80	.00
	6	.619	1.727	.03	.02	.00
	7	.552	1.828	.27	.05	.28

Collinearity Diagnostics[a]

Model	Dimension	Variance Proportions CntrSqrtWABCLInternal	CntrSqrtHABCLInternal	HsxWoInternalize	WsxHoInternalize
1	1				
	2				
	3				
2	1	.15	.16		
	2	.00	.00		
	3	.05	.01		
	4	.20	.10		
	5	.59	.73		
3	1	.12	.12	.01	.07
	2	.01	.01	.20	.06
	3	.01	.01	.32	.19
	4	.02	.00	.13	.21
	5	.17	.09	.01	.01
	6	.67	.52	.01	.05
	7	.00	.24	.32	.42

a. Dependent Variable: CplDASODA

Residuals Statistics[a]

	Minimum	Maximum	Mean	Std. Deviation	N
Predicted Value	36.2757	115.9203	73.5149	14.85887	101
Residual	-36.89735	33.72427	.00000	16.36785	101
Std. Predicted Value	-2.506	2.854	.000	1.000	101
Std. Residual	-2.186	1.998	.000	.970	101

a. Dependent Variable: CplDASODA

Charts

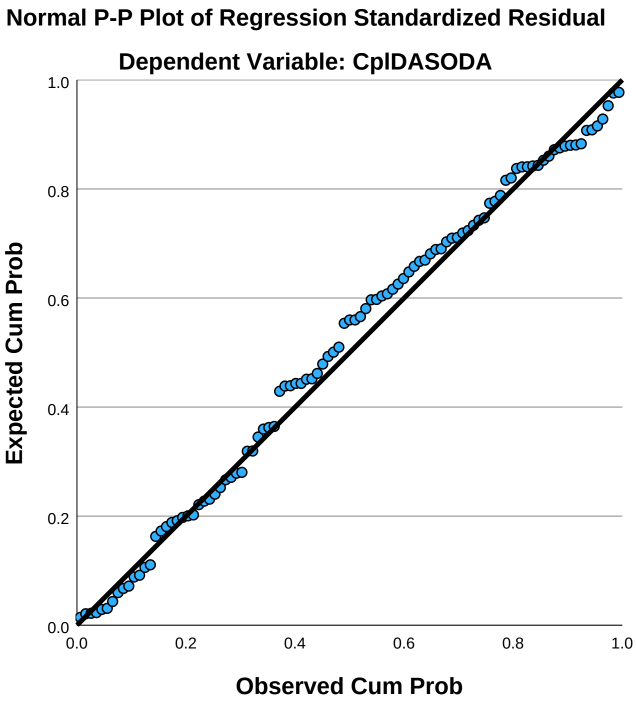

Regression

Notes

Output Created		20-DEC-2023 16:43:44
Comments		
Input	Data	C: \Users\jdwir\OneDrive\Marvin Research\DATA Sets\DyadicData_Whole Lot_122 (2023).sav
	Active Dataset	DataSet1
	Filter	<none>
	Weight	<none>
	Split File	<none>
	N of Rows in Working Data File	101
Missing Value Handling	Definition of Missing	User-defined missing values are treated as missing.
	Cases Used	Statistics are based on cases with no missing values for any variable used.
Syntax		REGRESSION<br>/DESCRIPTIVES MEAN STDDEV CORR SIG N<br>/MISSING LISTWISE<br>/STATISTICS COEFF OUTS R ANOVA COLLIN TOL CHANGE<br>/CRITERIA=PIN(.05) POUT(.10) TOLERANCE(.0001)<br>/NOORIGIN<br>/DEPENDENT CplDASODA<br>/METHOD=ENTER CntrSqrtWASRExternal CntrSqrtASRExternal<br>/METHOD=ENTER CntrSqrtHABCLExternal CntrSqrtWABCLExternal<br>/METHOD=ENTER HsxWoExternlize WsxHoExternalize<br>/RESIDUALS NORMPROB(ZRESID).
Resources	Processor Time	00:00:00.22
	Elapsed Time	00:00:00.17

Notes

	Memory Required	22848 bytes
	Additional Memory Required for Residual Plots	216 bytes

Descriptive Statistics

	Mean	Std. Deviation	N
CplDASODA	73.5149	22.10639	101
CntrSqrtWASRExternal	.0000	.76579	101
CntrSqrtASRExternal	.0000	.66642	101
CntrSqrtHABCLExternal	.0000	1.05887	101
CntrSqrtWABCLExternal	.0000	1.04202	101
HsxWoExternlize	-.0507	.68216	101
WsxHoExternalize	-.0065	.89337	101

Correlations

		CplDASODA	CntrSqrtWASRExternal	CntrSqrtASRExternal
Pearson Correlation	CplDASODA	1.000	-.078	.115
	CntrSqrtWASRExternal	-.078	1.000	.132
	CntrSqrtASRExternal	.115	.132	1.000
	CntrSqrtHABCLExternal	-.736	-.008	-.164
	CntrSqrtWABCLExternal	-.629	.062	-.074
	HsxWoExternlize	.063	-.078	.087
	WsxHoExternalize	.183	-.126	-.075
Sig. (1-tailed)	CplDASODA	.	.220	.126
	CntrSqrtWASRExternal	.220	.	.095
	CntrSqrtASRExternal	.126	.095	.
	CntrSqrtHABCLExternal	.000	.468	.050
	CntrSqrtWABCLExternal	.000	.268	.232
	HsxWoExternlize	.267	.219	.194
	WsxHoExternalize	.033	.104	.227
N	CplDASODA	101	101	101
	CntrSqrtWASRExternal	101	101	101
	CntrSqrtASRExternal	101	101	101
	CntrSqrtHABCLExternal	101	101	101
	CntrSqrtWABCLExternal	101	101	101
	HsxWoExternlize	101	101	101
	WsxHoExternalize	101	101	101

Correlations

		CntrSqrtHABCL External	CntrSqrtWABCL External	HsxWoExternlize
Pearson Correlation	CplDASODA	-.736	-.629	.063
	CntrSqrtWASRExternal	-.008	.062	-.078
	CntrSqrtASRExternal	-.164	-.074	.087
	CntrSqrtHABCLExternal	1.000	.630	-.091
	CntrSqrtWABCLExternal	.630	1.000	-.167
	HsxWoExternlize	-.091	-.167	1.000
	WsxHoExternalize	-.229	-.219	.023
Sig. (1-tailed)	CplDASODA	<.001	<.001	.267
	CntrSqrtWASRExternal	.468	.268	.219
	CntrSqrtASRExternal	.050	.232	.194
	CntrSqrtHABCLExternal	.	.000	.182
	CntrSqrtWABCLExternal	.000	.	.048
	HsxWoExternlize	.182	.048	.
	WsxHoExternalize	.011	.014	.410
N	CplDASODA	101	101	101
	CntrSqrtWASRExternal	101	101	101
	CntrSqrtASRExternal	101	101	101
	CntrSqrtHABCLExternal	101	101	101
	CntrSqrtWABCLExternal	101	101	101
	HsxWoExternlize	101	101	101
	WsxHoExternalize	101	101	101

Correlations

		WsxHoExternalize
Pearson Correlation	CplDASODA	.183
	CntrSqrtWASRExternal	-.126
	CntrSqrtASRExternal	-.075
	CntrSqrtHABCLExternal	-.229
	CntrSqrtWABCLExternal	-.219
	HsxWoExternlize	.023
	WsxHoExternalize	1.000
Sig. (1-tailed)	CplDASODA	.033
	CntrSqrtWASRExternal	.104
	CntrSqrtASRExternal	.227
	CntrSqrtHABCLExternal	.011
	CntrSqrtWABCLExternal	.014
	HsxWoExternlize	.410
	WsxHoExternalize	.
N	CplDASODA	101
	CntrSqrtWASRExternal	101
	CntrSqrtASRExternal	101
	CntrSqrtHABCLExternal	101
	CntrSqrtWABCLExternal	101
	HsxWoExternlize	101
	WsxHoExternalize	101

Variables Entered/Removed[a]

Model	Variables Entered	Variables Removed	Method
1	CntrSqrtASRExternal, CntrSqrtWASRExternal[b]	.	Enter
2	CntrSqrtWABCLExternal, CntrSqrtHABCLExternal[b]	.	Enter
3	HsxWoExternlize, WsxHoExternalize[b]	.	Enter

a. Dependent Variable: CplDASODA

b. All requested variables entered.

Model Summary[d]

Model	R	R Square	Adjusted R Square	Std. Error of the Estimate	Change Statistics: R Square Change	F Change	df1
1	.148[a]	.022	.002	22.08425	.022	1.100	2
2	.769[b]	.592	.575	14.41836	.570	66.956	2
3	.770[c]	.593	.568	14.53788	.002	.214	2

Model Summary[d]

Model	Change Statistics: df2	Sig. F Change
1	98	.337
2	96	<.001
3	94	.808

a. Predictors: (Constant), CntrSqrtASRExternal, CntrSqrtWASRExternal

b. Predictors: (Constant), CntrSqrtASRExternal, CntrSqrtWASRExternal, CntrSqrtWABCLExternal, CntrSqrtHABCLExternal

c. Predictors: (Constant), CntrSqrtASRExternal, CntrSqrtWASRExternal, CntrSqrtWABCLExternal, CntrSqrtHABCLExternal, HsxWoExternlize, WsxHoExternalize

d. Dependent Variable: CplDASODA

ANOVA[a]

Model		Sum of Squares	df	Mean Square	F	Sig.
1	Regression	1073.245	2	536.622	1.100	.337[b]
	Residual	47795.983	98	487.714		
	Total	48869.228	100			
2	Regression	28911.881	4	7227.970	34.768	<.001[c]
	Residual	19957.347	96	207.889		
	Total	48869.228	100			
3	Regression	29002.340	6	4833.723	22.871	<.001[d]
	Residual	19866.888	94	211.350		
	Total	48869.228	100			

a. Dependent Variable: CplDASODA

b. Predictors: (Constant), CntrSqrtASRExternal, CntrSqrtWASRExternal

c. Predictors: (Constant), CntrSqrtASRExternal, CntrSqrtWASRExternal, CntrSqrtWABCLExternal, CntrSqrtHABCLExternal

d. Predictors: (Constant), CntrSqrtASRExternal, CntrSqrtWASRExternal, CntrSqrtWABCLExternal, CntrSqrtHABCLExternal, HsxWoExternlize, ...

Coefficients[a]

Model		Unstandardized Coefficients B	Std. Error	Standardized Coefficients Beta	t	Sig.
1	(Constant)	73.515	2.197		33.454	<.001
	CntrSqrtWASRExternal	-2.724	2.909	-.094	-.936	.351
	CntrSqrtASRExternal	4.225	3.343	.127	1.264	.209
2	(Constant)	73.515	1.435		51.242	<.001
	CntrSqrtWASRExternal	-1.933	1.906	-.067	-1.014	.313
	CntrSqrtASRExternal	.361	2.214	.011	.163	.871
	CntrSqrtHABCLExternal	-11.831	1.775	-.567	-6.666	<.001
	CntrSqrtWABCLExternal	-5.667	1.788	-.267	-3.169	.002
3	(Constant)	73.445	1.451		50.623	<.001
	CntrSqrtWASRExternal	-2.073	1.940	-.072	-1.069	.288
	CntrSqrtASRExternal	.437	2.252	.013	.194	.846
	CntrSqrtHABCLExternal	-11.856	1.809	-.568	-6.556	<.001
	CntrSqrtWABCLExternal	-5.856	1.828	-.276	-3.204	.002
	HsxWoExternlize	-1.351	2.176	-.042	-.621	.536
	WsxHoExternalize	-.362	1.702	-.015	-.213	.832

Coefficients[a]

Model		Collinearity Statistics Tolerance	VIF
1	(Constant)		
	CntrSqrtWASRExternal	.983	1.018
	CntrSqrtASRExternal	.983	1.018
2	(Constant)		
	CntrSqrtWASRExternal	.976	1.025
	CntrSqrtASRExternal	.955	1.047
	CntrSqrtHABCLExternal	.589	1.699
	CntrSqrtWABCLExternal	.599	1.671
3	(Constant)		
	CntrSqrtWASRExternal	.958	1.044
	CntrSqrtASRExternal	.938	1.066
	CntrSqrtHABCLExternal	.576	1.735
	CntrSqrtWABCLExternal	.583	1.716
	HsxWoExternlize	.960	1.042
	WsxHoExternalize	.914	1.094

a. Dependent Variable: CplDASODA

Excluded Variables[a]

Model		Beta In	t	Sig.	Partial Correlation	Collinearity .
						Tolerance
1	CntrSqrtHABCLExternal	-.736[b]	-10.645	<.001	-.734	.973
	CntrSqrtWABCLExternal	-.620[b]	-7.862	<.001	-.624	.989
	HsxWoExternlize	.045[b]	.444	.658	.045	.984
	WsxHoExternalize	.184[b]	1.849	.067	.185	.981
2	HsxWoExternlize	-.042[c]	-.622	.536	-.064	.960
	WsxHoExternalize	-.014[c]	-.206	.837	-.021	.915

Excluded Variables[a]

Model		Collinearity Statistics	
		VIF	Minimum Tolerance
1	CntrSqrtHABCLExternal	1.028	.956
	CntrSqrtWABCLExternal	1.011	.976
	HsxWoExternlize	1.016	.973
	WsxHoExternalize	1.020	.969
2	HsxWoExternlize	1.042	.587
	WsxHoExternalize	1.093	.577

a. Dependent Variable: CplDASODA

b. Predictors in the Model: (Constant), CntrSqrtASRExternal, CntrSqrtWASRExternal

c. Predictors in the Model: (Constant), CntrSqrtASRExternal, CntrSqrtWASRExternal, CntrSqrtWABCLExternal, CntrSqrtHABCLExternal

Collinearity Diagnostics[a]

Model	Dimension	Eigenvalue	Condition Index	Variance Proportions (Constant)	CntrSqrtWASREExternal	CntrSqrtASRExternal
1	1	1.132	1.000	.00	.43	.43
	2	1.000	1.064	1.00	.00	.00
	3	.868	1.142	.00	.57	.57
2	1	1.672	1.000	.00	.00	.03
	2	1.126	1.219	.00	.49	.34
	3	1.000	1.293	1.00	.00	.00
	4	.841	1.410	.00	.50	.60
	5	.362	2.150	.00	.01	.02
3	1	1.833	1.000	.00	.00	.01
	2	1.218	1.227	.00	.30	.32
	3	1.070	1.309	.43	.05	.01
	4	.951	1.388	.52	.15	.04
	5	.797	1.517	.02	.32	.56
	6	.776	1.537	.02	.17	.04
	7	.356	2.270	.00	.01	.03

Collinearity Diagnostics[a]

Model	Dimension	Variance Proportions CntrSqrtHABCLExternal	CntrSqrtWABCLExternal	HsxWoExternlize	WsxHoExternalize
1	1				
	2				
	3				
2	1	.17	.16		
	2	.00	.02		
	3	.00	.00		
	4	.01	.02		
	5	.82	.80		
3	1	.12	.13	.03	.06
	2	.01	.00	.00	.14
	3	.01	.00	.38	.02
	4	.01	.00	.24	.06
	5	.00	.02	.19	.05
	6	.04	.06	.14	.67
	7	.81	.78	.02	.00

a. Dependent Variable: CplDASODA

Residuals Statistics[a]

	Minimum	Maximum	Mean	Std. Deviation	N
Predicted Value	43.4025	111.4594	73.5149	17.03007	101
Residual	-29.34205	39.92162	.00000	14.09499	101
Std. Predicted Value	-1.768	2.228	.000	1.000	101
Std. Residual	-2.018	2.746	.000	.970	101

a. Dependent Variable: CplDASODA

Charts

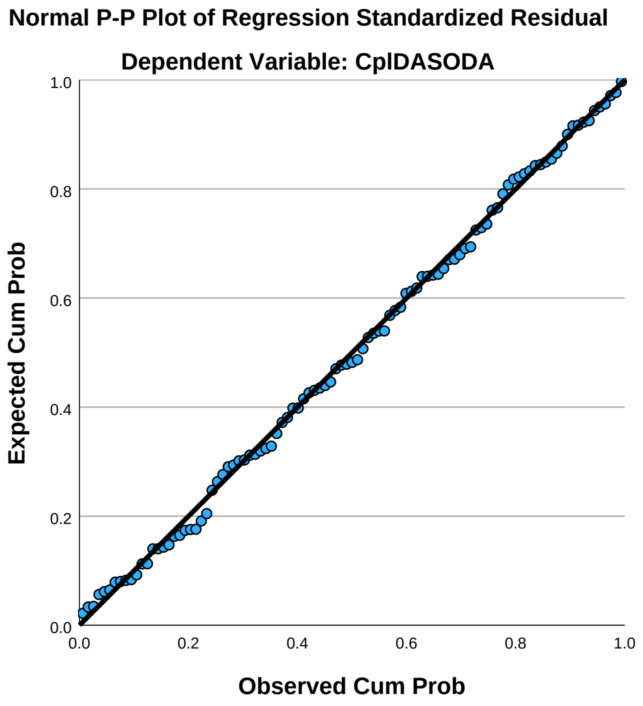

Supplement: Supplementary file 2 [file Data_Sheet_2.PDF]
