# Supplementary material for: Assessing self–other agreement and dyadic adjustment in marital dyads
Source: Front Psychol. 2024 Nov 15;15:1363165. doi: 10.3389/fpsyg.2024.1363165 (PMC11604461; doi:10.3389/fpsyg.2024.1363165)

Regression

Notes

Output Created		20-DEC-2023 16:33:35
Comments		
Input	Data	C:\Users\jdwir\OneDrive\Marvin Research\DATA Sets\DyadicData_WholeLot_122 (2023).sav
	Active Dataset	DataSet1
	Filter	<none>
	Weight	<none>
	Split File	<none>
	N of Rows in Working Data File	101
Missing Value Handling	Definition of Missing	User-defined missing values are treated as missing.
	Cases Used	Statistics are based on cases with no missing values for any variable used.
Syntax		REGRESSION<br>/DESCRIPTIVES MEAN STDDEV CORR SIG N<br>/MISSING LISTWISE<br>/STATISTICS COEFF OUTS R ANOVA COLLIN TOL CHANGE<br>/CRITERIA=PIN(.05) POUT(.10) TOLERANCE(.0001)<br>/NOORIGIN<br>/DEPENDENT CplDASODA<br>/METHOD=ENTER CntrSqrtWASRTTLProb CntrSqrtHASRTTLProb<br>/METHOD=ENTER CntrSqrtHABCLTotProb CntrSqrtWABCLTTLProb<br>/METHOD=ENTER HsxWo_InteractionTTLProb WsxHo_InteractionTTLProb<br>/RESIDUALS NORMPROB(ZRESID).

Notes

Resources	Processor Time	00:00:00.16
	Elapsed Time	00:00:00.14
	Memory Required	22848 bytes
	Additional Memory Required for Residual Plots	216 bytes

Descriptive Statistics

	Mean	Std. Deviation	N
CplDASODA	73.5149	22.10639	101
CntrSqrtWASRTTLProb	.0000	.81344	101
CntrSqrtHASRTTLProb	.0000	.78104	101
CntrSqrtHABCLTotProb	.0000	.96540	101
CntrSqrtWABCLTTLProb	.0000	.97355	101
HsxWo_InteractionTTLProb	.1069	.81420	101
WsxHo_InteractionTTLProb	.1403	.88107	101

Correlations

		CplDASODA	CntrSqrtWASRTTLProb	CntrSqrtHASRTTLProb
Pearson Correlation	CplDASODA	1.000	-.098	.039
	CntrSqrtWASRTTLProb	-.098	1.000	.125
	CntrSqrtHASRTTLProb	.039	.125	1.000
	CntrSqrtHABCLTotProb	-.649	.180	-.011
	CntrSqrtWABCLTTLProb	-.570	.183	.142
	HsxWo_InteractionTTLProb	.146	-.097	.086
	WsxHo_InteractionTTLProb	.341	-.122	-.073
Sig. (1-tailed)	CplDASODA	.	.164	.350
	CntrSqrtWASRTTLProb	.164	.	.106
	CntrSqrtHASRTTLProb	.350	.106	.
	CntrSqrtHABCLTotProb	.000	.035	.457
	CntrSqrtWABCLTTLProb	.000	.034	.078
	HsxWo_InteractionTTLProb	.072	.167	.196
	WsxHo_InteractionTTLProb	.000	.112	.233
N	CplDASODA	101	101	101
	CntrSqrtWASRTTLProb	101	101	101

Correlations

		CntrSqrtHABCLTotProb	CntrSqrtWABCLTTLProb	HsxWo_InteractionTTLProb
Pearson Correlation	CplDASODA	-.649	-.570	.146
	CntrSqrtWASRTTLProb	.180	.183	-.097
	CntrSqrtHASRTTLProb	-.011	.142	.086
	CntrSqrtHABCLTotProb	1.000	.519	-.205
	CntrSqrtWABCLTTLProb	.519	1.000	-.141
	HsxWo_InteractionTTLProb	-.205	-.141	1.000
	WsxHo_InteractionTTLProb	-.482	-.317	.156
Sig. (1-tailed)	CplDASODA	<.001	<.001	.072
	CntrSqrtWASRTTLProb	.035	.034	.167
	CntrSqrtHASRTTLProb	.457	.078	.196
	CntrSqrtHABCLTotProb	.	.000	.020
	CntrSqrtWABCLTTLProb	.000	.	.079
	HsxWo_InteractionTTLProb	.020	.079	.
	WsxHo_InteractionTTLProb	.000	.001	.059
N	CplDASODA	101	101	101
	CntrSqrtWASRTTLProb	101	101	101

Correlations

		WsxHo_InteractionTTLProb
Pearson Correlation	CplDASODA	.341
	CntrSqrtWASRTTLProb	-.122
	CntrSqrtHASRTTLProb	-.073
	CntrSqrtHABCLTotProb	-.482
	CntrSqrtWABCLTTLProb	-.317
	HsxWo_InteractionTTLProb	.156
	WsxHo_InteractionTTLProb	1.000
Sig. (1-tailed)	CplDASODA	<.001
	CntrSqrtWASRTTLProb	.112
	CntrSqrtHASRTTLProb	.233
	CntrSqrtHABCLTotProb	.000
	CntrSqrtWABCLTTLProb	.001
	HsxWo_InteractionTTLProb	.059
	WsxHo_InteractionTTLProb	.
N	CplDASODA	101
	CntrSqrtWASRTTLProb	101

Correlations

	CplDASODA	CntrSqrtWASRTTLProb	CntrSqrtHASRTTLProb
CntrSqrtHASRTTLProb	101	101	101
CntrSqrtHABCLTotProb	101	101	101
CntrSqrtWABCLTTLProb	101	101	101
HsxWo_InteractionTTLProb	101	101	101
WsxHo_InteractionTTLProb	101	101	101

Correlations

	CntrSqrtHABCLTotProb	CntrSqrtWABCLTTLProb	HsxWo_InteractionTTLProb
CntrSqrtHASRTTLProb	101	101	101
CntrSqrtHABCLTotProb	101	101	101
CntrSqrtWABCLTTLProb	101	101	101
HsxWo_InteractionTTLProb	101	101	101
WsxHo_InteractionTTLProb	101	101	101

Correlations

	WsxHo_InteractionTTLProb
CntrSqrtHASRTTLProb	101
CntrSqrtHABCLTotProb	101
CntrSqrtWABCLTTLProb	101
HsxWo_InteractionTTLProb	101
WsxHo_InteractionTTLProb	101

Variables Entered/Removed[a]

Model	Variables Entered	Variables Removed	Method
1	CntrSqrtHASRTTLProb, CntrSqrtWASRTTLProb[b]	.	Enter
2	CntrSqrtHABCLTotProb, CntrSqrtWABCLTTLProb[b]	.	Enter
3	HsxWo_InteractionTTLProb, WsxHo_InteractionTTLProb[b]	.	Enter

a. Dependent Variable: CplDASODA

b. All requested variables entered.

Model Summary[d]

Model	R	R Square	Adjusted R Square	Std. Error of the Estimate	Change Statistics		
					R Square Change	F Change	df1
1	.111[a]	.012	-.008	22.19230	.012	.614	2
2	.709[b]	.503	.482	15.90410	.491	47.407	2
3	.709[c]	.503	.472	16.06866	.000	.022	2

Model Summary[d]

Model	Change Statistics	
	df2	Sig. F Change
1	98	.543
2	96	<.001
3	94	.978

a. Predictors: (Constant), CntrSqrtHASRTTLProb, CntrSqrtWASRTTLProb

b. Predictors: (Constant), CntrSqrtHASRTTLProb, CntrSqrtWASRTTLProb, CntrSqrtHABCLTotProb, CntrSqrtWABCLTTLProb

c. Predictors: (Constant), CntrSqrtHASRTTLProb, CntrSqrtWASRTTLProb, CntrSqrtHABCLTotProb, CntrSqrtWABCLTTLProb, HsxWo_InteractionTTLProb, WsxHo_InteractionTTLProb

d. Dependent Variable: CplDASODA

ANOVA[a]

Model		Sum of Squares	df	Mean Square	F	Sig.
1	Regression	604.387	2	302.193	.614	.543[b]
	Residual	48264.841	98	492.498		
	Total	48869.228	100			
2	Regression	24586.935	4	6146.734	24.301	<.001[c]
	Residual	24282.293	96	252.941		
	Total	48869.228	100			
3	Regression	24598.270	6	4099.712	15.878	<.001[d]
	Residual	24270.958	94	258.202		
	Total	48869.228	100			

a. Dependent Variable: CplDASODA

b. Predictors: (Constant), CntrSqrtHASRTTLProb, CntrSqrtWASRTTLProb

c. Predictors: (Constant), CntrSqrtHASRTTLProb, CntrSqrtWASRTTLProb, CntrSqrtHABCLTotProb, CntrSqrtWABCLTTLProb

d. Predictors: (Constant), CntrSqrtHASRTTLProb, CntrSqrtWASRTTLProb, CntrSqrtHABCLTotProb, CntrSqrtWABCLTTLProb, HsxWo_InteractionTTLProb, WsxHo_InteractionTTLProb

Coefficients[a]

Model		Unstandardized Coefficients B	Unstandardized Coefficients Std. Error	Standardized Coefficients Beta	t	Sig.
1	(Constant)	73.515	2.208		33.292	<.001
	CntrSqrtWASRTTLProb	-2.854	2.750	-.105	-1.038	.302
	CntrSqrtHASRTTLProb	1.475	2.864	.052	.515	.608
2	(Constant)	73.515	1.583		46.454	<.001
	CntrSqrtWASRTTLProb	1.098	2.012	.040	.546	.587
	CntrSqrtHASRTTLProb	2.176	2.081	.077	1.046	.298
	CntrSqrtHABCLTotProb	-10.967	1.950	-.479	-5.624	<.001
	CntrSqrtWABCLTTLProb	-7.707	1.948	-.339	-3.956	<.001
3	(Constant)	73.466	1.638		44.860	<.001
	CntrSqrtWASRTTLProb	1.100	2.038	.040	.540	.591
	CntrSqrtHASRTTLProb	2.219	2.120	.078	1.047	.298
	CntrSqrtHABCLTotProb	-10.808	2.154	-.472	-5.019	<.001
	CntrSqrtWABCLTTLProb	-7.683	1.975	-.338	-3.889	<.001
	HsxWo_InteractionTTLProb	-.109	2.037	-.004	-.054	.957
	WsxHo_InteractionTTLProb	.433	2.102	.017	.206	.837

Coefficients[a]

Model		Collinearity Statistics Tolerance	Collinearity Statistics VIF
1	(Constant)		
	CntrSqrtWASRTTLProb	.984	1.016
	CntrSqrtHASRTTLProb	.984	1.016
2	(Constant)		
	CntrSqrtWASRTTLProb	.944	1.059
	CntrSqrtHASRTTLProb	.958	1.044
	CntrSqrtHABCLTotProb	.714	1.401
	CntrSqrtWABCLTTLProb	.703	1.422
3	(Constant)		
	CntrSqrtWASRTTLProb	.939	1.065
	CntrSqrtHASRTTLProb	.942	1.062
	CntrSqrtHABCLTotProb	.597	1.674
	CntrSqrtWABCLTTLProb	.698	1.432
	HsxWo_InteractionTTLProb	.938	1.066
	WsxHo_InteractionTTLProb	.753	1.328

a. Dependent Variable: CplDASODA

Excluded Variables[a]

Model		Beta In	t	Sig.	Partial Correlation	Collinearity . Tolerance
1	CntrSqrtHABCLTotProb	-.651[b]	-8.293	<.001	-.644	.966
	CntrSqrtWABCLTTLProb	-.586[b]	-6.929	<.001	-.575	.952
	HsxWo_InteractionTTLProb	.134[b]	1.328	.187	.134	.981
	WsxHo_InteractionTTLProb	.338[b]	3.525	<.001	.337	.982
2	HsxWo_InteractionTTLProb	-.003[c]	-.040	.969	-.004	.943
	WsxHo_InteractionTTLProb	.017[c]	.204	.839	.021	.757

Excluded Variables[a]

Model		Collinearity Statistics	
		VIF	Minimum Tolerance
1	CntrSqrtHABCLTotProb	1.035	.951
	CntrSqrtWABCLTTLProb	1.050	.952
	HsxWo_InteractionTTLProb	1.020	.973
	WsxHo_InteractionTTLProb	1.019	.971
2	HsxWo_InteractionTTLProb	1.060	.700
	WsxHo_InteractionTTLProb	1.322	.603

a. Dependent Variable: CplDASODA

b. Predictors in the Model: (Constant), CntrSqrtHASRTTLProb, CntrSqrtWASRTTLProb

c. Predictors in the Model: (Constant), CntrSqrtHASRTTLProb, CntrSqrtWASRTTLProb, CntrSqrtHABCLTotProb, CntrSqrtWABCLTTLProb

Collinearity Diagnostics[a]

Model	Dimension	Eigenvalue	Condition Index	Variance Proportions (Constant)	CntrSqrtWASRTTLProb	CntrSqrtHASRTTLProb
1	1	1.125	1.000	.00	.44	.44
	2	1.000	1.061	1.00	.00	.00
	3	.875	1.134	.00	.56	.56
2	1	1.653	1.000	.00	.09	.02
	2	1.037	1.263	.00	.12	.65
	3	1.000	1.286	1.00	.00	.00
	4	.852	1.393	.00	.78	.23
	5	.458	1.900	.00	.00	.09
3	1	2.079	1.000	.01	.03	.00
	2	1.166	1.335	.20	.08	.30
	3	1.020	1.427	.49	.04	.30
	4	.918	1.505	.03	.59	.02
	5	.769	1.644	.09	.25	.28
	6	.642	1.799	.16	.01	.02
	7	.406	2.263	.03	.00	.08

Collinearity Diagnostics[a]

Model	Dimension	Variance Proportions CntrSqrtHABCLTotProb	CntrSqrtWABCLTTLProb	HsxWo_InteractionTTLProb	WsxHo_InteractionTTLProb
1	1				
	2				
	3				
2	1	.17	.18		
	2	.09	.01		
	3	.00	.00		
	4	.01	.06		
	5	.73	.75		
3	1	.09	.09	.04	.09
	2	.00	.03	.19	.01
	3	.05	.01	.00	.00
	4	.01	.02	.24	.08
	5	.01	.01	.53	.02
	6	.00	.45	.01	.52
	7	.84	.40	.00	.27

a. Dependent Variable: CplDASODA

Residuals Statistics[a]

	Minimum	Maximum	Mean	Std. Deviation	N
Predicted Value	38.0919	107.8242	73.5149	15.68384	101
Residual	-33.02824	40.90814	.00000	15.57914	101
Std. Predicted Value	-2.259	2.188	.000	1.000	101
Std. Residual	-2.055	2.546	.000	.970	101

a. Dependent Variable: CplDASODA

Charts

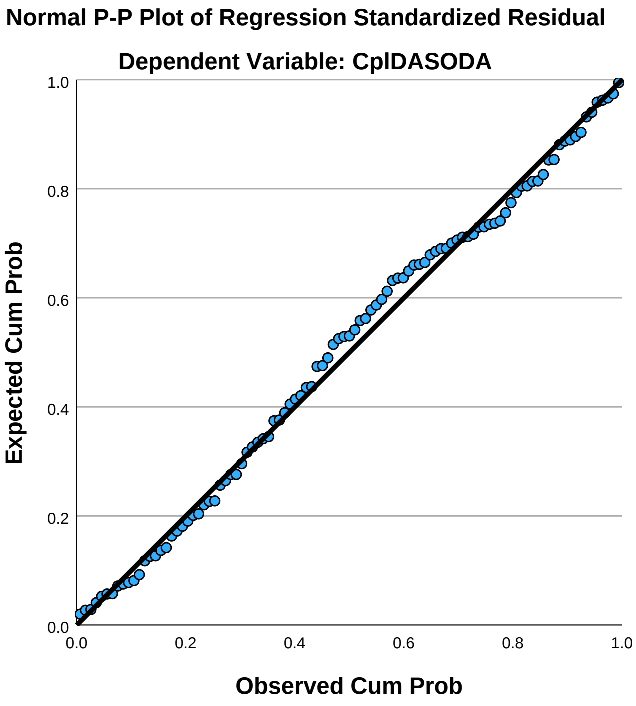

Supplement: Supplementary file 3 [file Data_Sheet_3.PDF]
