# Supplementary material for: Assessing self–other agreement and dyadic adjustment in marital dyads
Source: Front Psychol. 2024 Nov 15;15:1363165. doi: 10.3389/fpsyg.2024.1363165 (PMC11604461; doi:10.3389/fpsyg.2024.1363165)
Supplement: Supplementary file 4 [file Data_Sheet_4.PDF]

Univariate Analysis of Variance

Notes

Output Created		20-DEC-2023 11:41:48
Comments		
Input	Data	C: \Users\jdwir\OneDrive\Marvin Research\DATA Sets\DyadicData_WholeLot_122 (2023).sav
	Active Dataset	DataSet1
	Filter	<none>
	Weight	<none>
	Split File	<none>
	N of Rows in Working Data File	101
Missing Value Handling	Definition of Missing	User-defined missing values are treated as missing.
	Cases Used	Statistics are based on all cases with valid data for all variables in the model.
Syntax		UNIANOVA CntrSqrtCplInTPDI BY GroupAssociation WITH YearsEd Age YearsEdWif AgeWif<br>/METHOD=SSTYPE(3)<br>/INTERCEPT=INCLUDE<br>/PLOT=PROFILE (GroupAssociation) TYPE=LINE ERRORBAR=CI MEANREFERENCE=NO YAXIS=AUTO<br>/EMMEANS=TABLES (GroupAssociation) WITH (YearsEd=MEAN Age=MEAN YearsEdWif=MEAN AgeWif=MEAN) COMPARE<br>ADJ(BONFERRONI)<br>/PRINT ETASQ DESCRIPTIVE HOMOGENEITY OPOWER<br>/CRITERIA=ALPHA(.05)<br>/DESIGN=YearsEd Age ...

Notes

Resources	Processor Time	00:00:00.17
	Elapsed Time	00:00:00.15

Between-Subjects Factors

		Value Label	N
Group Association	1.00	Medical	20
	2.00	Unhappy	61
	3.00	Happy	20

Descriptive Statistics

Dependent Variable: CntrSqrtCplInTPDI

Group Association	Mean	Std. Deviation	N
Medical	-1.4033	2.06165	20
Unhappy	1.1948	2.05778	61
Happy	-2.2409	1.26163	20
Total	.0000	2.43303	101

Levene's Test of Equality of Error Variances[a]

Dependent Variable: CntrSqrtCplInTPDI

F	df1	df2	Sig.
1.763	2	98	.177

Tests the null hypothesis that the error variance of the dependent variable is equal across groups.

a. Design: Intercept + YearsEd + Age + YearsEdWif + AgeWif + ...

Tests of Between-Subjects Effects

Dependent Variable: CntrSqrtCplInTPDI

Source	Type III Sum of Squares	df	Mean Square	F	Sig.	Partial Eta Squared
Corrected Model	240.570[a]	6	40.095	10.726	<.001	.406
Intercept	3.067	1	3.067	.821	.367	.009
YearsEd	.950	1	.950	.254	.615	.003
Age	1.616	1	1.616	.432	.512	.005
YearsEdWif	3.774	1	3.774	1.010	.318	.011
AgeWif	6.141	1	6.141	1.643	.203	.017
GroupAssociation	83.865	2	41.933	11.217	<.001	.193
Error	351.396	94	3.738			
Total	591.965	101				
Corrected Total	591.965	100				

Tests of Between-Subjects Effects

Dependent Variable: CntrSqrtCplInTPDI

Source	Noncent. Parameter	Observed Power[b]
Corrected Model	64.354	1.000
Intercept	.821	.146
YearsEd	.254	.079
Age	.432	.100
YearsEdWif	1.010	.169
AgeWif	1.643	.245
GroupAssociation	22.434	.991
Error		
Total		
Corrected Total		

a. R Squared = .406 (Adjusted R Squared = .369)

b. Computed using alpha = .05

Estimated Marginal Means

Group Association

Estimates

Dependent Variable: CntrSqrtCplInTPDI

Group Association	Mean	Std. Error	95% Confidence Interval Lower Bound	95% Confidence Interval Upper Bound
Medical	-1.089[a]	.497	-2.075	-.103
Unhappy	.983[a]	.289	.409	1.557
Happy	-1.910[a]	.497	-2.897	-.924

a. Covariates appearing in the model are evaluated at the following values: HYears of Education = 15.1188, HAge of Participant = 42.6535, WYears of Education = 11.5248, WAge of Participant = ...

Pairwise Comparisons

Dependent Variable: CntrSqrtCplInTPDI

(I) Group Association	(J) Group Association	Mean Difference (I-J)	Std. Error	Sig.[b]	95% Confidence Interval for ...[b] Lower Bound
Medical	Unhappy	-2.072*	.631	.004	-3.611
	Happy	.821	.637	.602	-.732
Unhappy	Medical	2.072*	.631	.004	.534
	Happy	2.894*	.631	<.001	1.355
Happy	Medical	-.821	.637	.602	-2.375
	Unhappy	-2.894*	.631	<.001	-4.432

Pairwise Comparisons

Dependent Variable: CntrSqrtCplInTPDI

(I) Group Association	(J) Group Association	95% Confidence Interval for ...[b] Upper Bound
Medical	Unhappy	-.534
	Happy	2.375
Unhappy	Medical	3.611
	Happy	4.432
Happy	Medical	.732
	Unhappy	-1.355

Based on estimated marginal means

*. The mean difference is significant at the .05 level.

b. Adjustment for multiple comparisons: Bonferroni.

Univariate Tests

Dependent Variable: CntrSqrtCplInTPDI

	Sum of Squares	df	Mean Square	F	Sig.	Partial Eta Squared
Contrast	83.865	2	41.933	11.217	<.001	.193
Error	351.396	94	3.738			

Univariate Tests

Dependent Variable: CntrSqrtCplInTPDI

	Noncent. Parameter	Observed Power[a]
Contrast	22.434	.991
Error		

The F tests the effect of Group Association. This test is based on the linearly independent pairwise comparisons among the estimated marginal means.

a. Computed using alpha = .05

Profile Plots

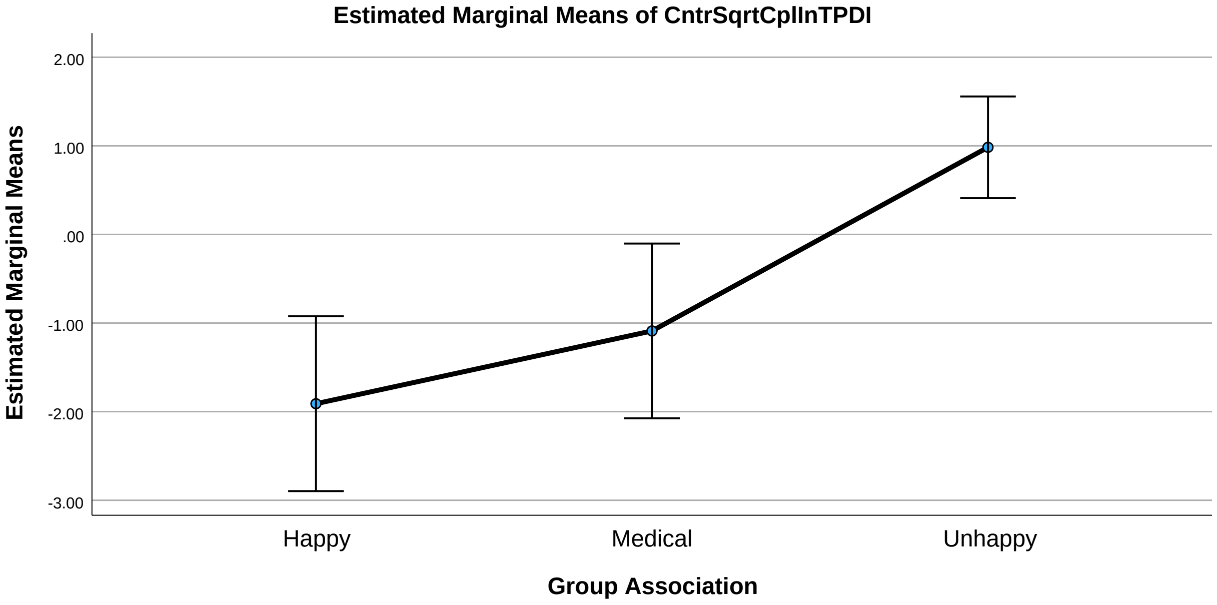

Covariates appearing in the model are evaluated at the following values: HYears of Education = 15.1188, HAge of Participant = 42.6535, WYears of Education = 11.5248, WAge of Participant = 41.2574

Error bars: 95% CI

Univariate Analysis of Variance

Notes

Output Created		20-DEC-2023 12:02:21
Comments		
Input	Data	C: \Users\jdwir\OneDrive\Marvin Research\DATA Sets\DyadicData_Whole Lot_122 (2023).sav
	Active Dataset	DataSet1
	Filter	<none>
	Weight	<none>
	Split File	<none>
	N of Rows in Working Data File	101
Missing Value Handling	Definition of Missing	User-defined missing values are treated as missing.
	Cases Used	Statistics are based on all cases with valid data for all variables in the model.
Syntax		UNIANOVA CntrSqrtCplExTPDI BY GroupAssociation WITH YearsEd Age YearsEdWif AgeWif<br>/METHOD=SSTYPE(3)<br>/INTERCEPT=INCLUDE<br>/PLOT=PROFILE (GroupAssociation) TYPE=LINE ERRORBAR=CI MEANREFERENCE=NO YAXIS=AUTO<br>/EMMEANS=TABLES (GroupAssociation) WITH (YearsEd=MEAN Age=MEAN YearsEdWif=MEAN AgeWif=MEAN) COMPARE<br>ADJ(BONFERRONI)<br>/PRINT ETASQ DESCRIPTIVE HOMOGENEITY OPOWER<br>/CRITERIA=ALPHA(.05)<br>/DESIGN=YearsEd Age ...
Resources	Processor Time	00:00:00.20
	Elapsed Time	00:00:00.14

Between-Subjects Factors

		Value Label	N
Group Association	1.00	Medical	20
	2.00	Unhappy	61
	3.00	Happy	20

Descriptive Statistics

Dependent Variable: CntrSqrtCplExTPDI

Group Association	Mean	Std. Deviation	N
Medical	-2.8366	2.27791	20
Unhappy	1.9678	2.28488	61
Happy	-3.1651	1.34507	20
Total	.0000	3.23064	101

Levene's Test of Equality of Error Variances[a]

Dependent Variable: CntrSqrtCplExTPDI

F	df1	df2	Sig.
1.518	2	98	.224

Tests the null hypothesis that the error variance of the dependent variable is equal across groups.

a. Design: Intercept + YearsEd + Age + YearsEdWif + AgeWif + ...

Tests of Between-Subjects Effects

Dependent Variable: CntrSqrtCplExTPDI

Source	Type III Sum of Squares	df	Mean Square	F	Sig.	Partial Eta Squared
Corrected Model	608.436[a]	6	101.406	21.900	<.001	.583
Intercept	.236	1	.236	.051	.822	.001
YearsEd	1.496	1	1.496	.323	.571	.003
Age	1.937	1	1.937	.418	.519	.004
YearsEdWif	5.417	1	5.417	1.170	.282	.012
AgeWif	1.665	1	1.665	.360	.550	.004
GroupAssociation	263.827	2	131.913	28.488	<.001	.377
Error	435.268	94	4.631			
Total	1043.704	101				
Corrected Total	1043.704	100				

Tests of Between-Subjects Effects

Dependent Variable: CntrSqrtCplExTPDI

Source	Noncent. Parameter	Observed Power[b]
Corrected Model	131.397	1.000
Intercept	.051	.056
YearsEd	.323	.087
Age	.418	.098
YearsEdWif	1.170	.188
AgeWif	.360	.091
GroupAssociation	56.976	1.000
Error		
Total		
Corrected Total		

a. R Squared = .583 (Adjusted R Squared = .556)

b. Computed using alpha = .05

Estimated Marginal Means

Group Association

Estimates

Dependent Variable: CntrSqrtCplExTPDI

Group Association	Mean	Std. Error	95% Confidence Interval Lower Bound	95% Confidence Interval Upper Bound
Medical	-2.693[a]	.553	-3.791	-1.596
Unhappy	1.812[a]	.322	1.173	2.451
Happy	-2.833[a]	.553	-3.931	-1.735

a. Covariates appearing in the model are evaluated at the following values: HYears of Education = 15.1188, HAge of Participant = 42.6535, WYears of Education = 11.5248, WAge of Participant = ...

Pairwise Comparisons

Dependent Variable: CntrSqrtCplExTPDI

(I) Group Association	(J) Group Association	Mean Difference (I-J)	Std. Error	Sig.[b]	95% Confidence Interval for ...[b] Lower Bound
Medical	Unhappy	-4.505*	.702	<.001	-6.217
	Happy	.139	.709	1.000	-1.590
Unhappy	Medical	4.505*	.702	<.001	2.793
	Happy	4.645*	.702	<.001	2.932
Happy	Medical	-.139	.709	1.000	-1.868
	Unhappy	-4.645*	.702	<.001	-6.357

Pairwise Comparisons

Dependent Variable: CntrSqrtCplExTPDI

(I) Group Association	(J) Group Association	95% Confidence Interval for ...[b] Upper Bound
Medical	Unhappy	-2.793
	Happy	1.868
Unhappy	Medical	6.217
	Happy	6.357
Happy	Medical	1.590
	Unhappy	-2.932

Based on estimated marginal means

*. The mean difference is significant at the .05 level.

b. Adjustment for multiple comparisons: Bonferroni.

Univariate Tests

Dependent Variable: CntrSqrtCplExTPDI

	Sum of Squares	df	Mean Square	F	Sig.	Partial Eta Squared
Contrast	263.827	2	131.913	28.488	<.001	.377
Error	435.268	94	4.631			

Univariate Tests

Dependent Variable: CntrSqrtCplExTPDI

	Noncent. Parameter	Observed Power[a]
Contrast	56.976	1.000
Error		

The F tests the effect of Group Association. This test is based on the linearly independent pairwise comparisons among the estimated marginal means.

a. Computed using alpha = .05

Profile Plots

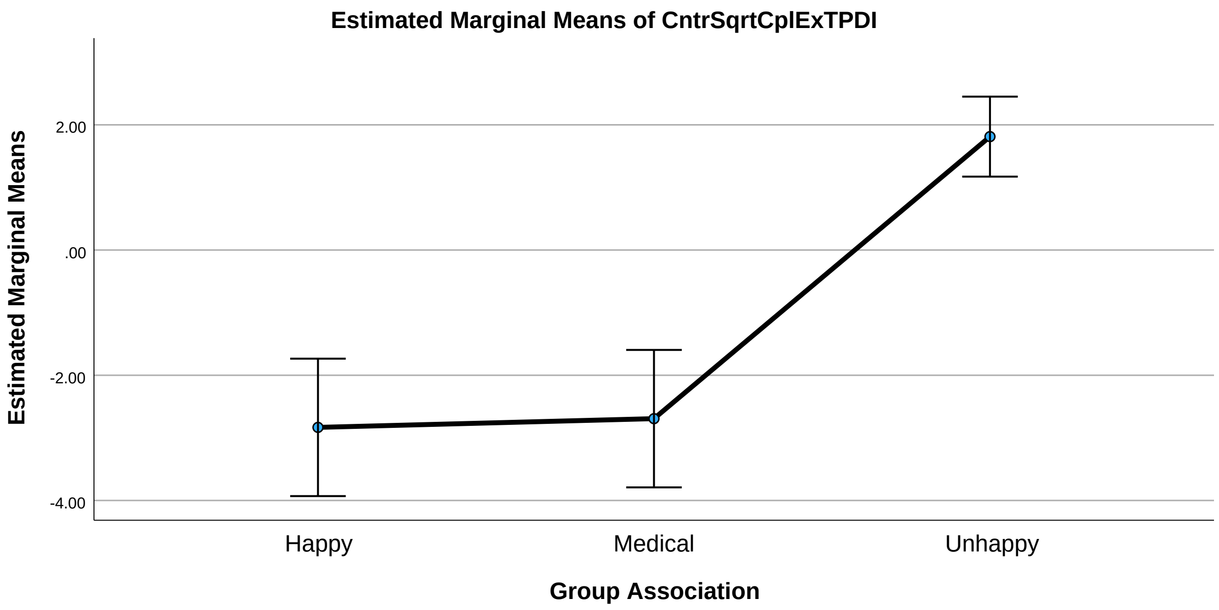

Covariates appearing in the model are evaluated at the following values: HYears of Education = 15.1188, HAge of Participant = 42.6535, WYears of Education = 11.5248, WAge of Participant = 41.2574

Error bars: 95% CI
