# Supplementary material for: Assessing self–other agreement and dyadic adjustment in marital dyads
Source: Front Psychol. 2024 Nov 15;15:1363165. doi: 10.3389/fpsyg.2024.1363165 (PMC11604461; doi:10.3389/fpsyg.2024.1363165)
Supplement: Supplementary file 5 [file Data_Sheet_5.PDF]

Univariate Analysis of Variance

Notes

Output Created		20-DEC-2023 11:13:42
Comments		
Input	Data	C:\Users\jdwir\OneDrive\Marvin Research\DATA Sets\DyadicData_WholeLot_122 (2023).sav
	Active Dataset	DataSet1
	Filter	<none>
	Weight	<none>
	Split File	<none>
	N of Rows in Working Data File	101
Missing Value Handling	Definition of Missing	User-defined missing values are treated as missing.
	Cases Used	Statistics are based on all cases with valid data for all variables in the model.
Syntax		UNIANOVA CntrSqrtCplTPDI BY GroupAssociation WITH YearsEd Age YearsEdWif AgeWif<br>/METHOD=SSTYPE(3)<br>/INTERCEPT=INCLUDE<br>/PLOT=PROFILE (GroupAssociation) TYPE=LINE ERRORBAR=CI MEANREFERENCE=NO YAXIS=AUTO<br>/EMMEANS=TABLES (GroupAssociation) WITH (YearsEd=MEAN Age=MEAN YearsEdWif=MEAN AgeWif=MEAN) COMPARE<br>ADJ(BONFERRONI)<br>/PRINT ETASQ DESCRIPTIVE HOMOGENEITY OPOWER<br>/CRITERIA=ALPHA(.05)<br>/DESIGN=YearsEd Age ...

Notes

Resources	Processor Time	00:00:00.20
	Elapsed Time	00:00:00.15

Between-Subjects Factors

		Value Label	N
Group Association	1.00	Medical	20
	2.00	Unhappy	61
	3.00	Happy	20

Descriptive Statistics

Dependent Variable: CntrSqrtCplTPDI

Group Association	Mean	Std. Deviation	N
Medical	-1.9293	1.86455	20
Unhappy	1.7015	2.28551	61
Happy	-3.2599	1.46392	20
Total	.0001	2.97282	101

Levene's Test of Equality of Error Variances[a]

Dependent Variable: CntrSqrtCplTPDI

F	df1	df2	Sig.
1.101	2	98	.337

Tests the null hypothesis that the error variance of the dependent variable is equal across groups.

a. Design: Intercept + YearsEd + Age + YearsEdWif + AgeWif + ...

Tests of Between-Subjects Effects

Dependent Variable: CntrSqrtCplTPDI

Source	Type III Sum of Squares	df	Mean Square	F	Sig.	Partial Eta Squared
Corrected Model	473.341[a]	6	78.890	18.068	<.001	.536
Intercept	.109	1	.109	.025	.875	.000
YearsEd	.173	1	.173	.040	.842	.000
Age	2.335	1	2.335	.535	.466	.006
YearsEdWif	5.001	1	5.001	1.145	.287	.012
AgeWif	3.717	1	3.717	.851	.359	.009
GroupAssociation	199.747	2	99.874	22.874	<.001	.327
Error	410.424	94	4.366			
Total	883.764	101				
Corrected Total	883.764	100				

Tests of Between-Subjects Effects

Dependent Variable: CntrSqrtCplTPDI

Source	Noncent. Parameter	Observed Power[b]
Corrected Model	108.410	1.000
Intercept	.025	.053
YearsEd	.040	.054
Age	.535	.112
YearsEdWif	1.145	.185
AgeWif	.851	.150
GroupAssociation	45.748	1.000
Error		
Total		
Corrected Total		

a. R Squared = .536 (Adjusted R Squared = .506)

b. Computed using alpha = .05

Estimated Marginal Means

Group Association

Estimates

Dependent Variable: CntrSqrtCplTPDI

Group Association	Mean	Std. Error	95% Confidence Interval Lower Bound	95% Confidence Interval Upper Bound
Medical	-1.707[a]	.537	-2.772	-.641
Unhappy	1.521[a]	.312	.901	2.142
Happy	-2.934[a]	.537	-4.000	-1.868

a. Covariates appearing in the model are evaluated at the following values: HYears of Education = 15.1188, HAge of Participant = 42.6535, WYears of Education = 11.5248, WAge of Participant = ...

Pairwise Comparisons

Dependent Variable: CntrSqrtCplTPDI

(I) Group Association	(J) Group Association	Mean Difference (I-J)	Std. Error	Sig.[b]	95% Confidence Interval for ...[b] Lower Bound
Medical	Unhappy	-3.228*	.682	<.001	-4.890
	Happy	1.227	.689	.234	-.452
Unhappy	Medical	3.228*	.682	<.001	1.566
	Happy	4.455*	.682	<.001	2.792
Happy	Medical	-1.227	.689	.234	-2.906
	Unhappy	-4.455*	.682	<.001	-6.118

Pairwise Comparisons

Dependent Variable: CntrSqrtCplTPDI

(I) Group Association	(J) Group Association	95% Confidence Interval for ...[b] Upper Bound
Medical	Unhappy	-1.566
	Happy	2.906
Unhappy	Medical	4.890
	Happy	6.118
Happy	Medical	.452
	Unhappy	-2.792

Based on estimated marginal means

*. The mean difference is significant at the .05 level.

b. Adjustment for multiple comparisons: Bonferroni.

Univariate Tests

Dependent Variable: CntrSqrtCplTPDI

	Sum of Squares	df	Mean Square	F	Sig.	Partial Eta Squared
Contrast	199.747	2	99.874	22.874	<.001	.327
Error	410.424	94	4.366			

Univariate Tests

Dependent Variable: CntrSqrtCplTPDI

	Noncent. Parameter	Observed Power[a]
Contrast	45.748	1.000
Error		

The F tests the effect of Group Association. This test is based on the linearly independent pairwise comparisons among the estimated marginal means.

a. Computed using alpha = .05

Profile Plots

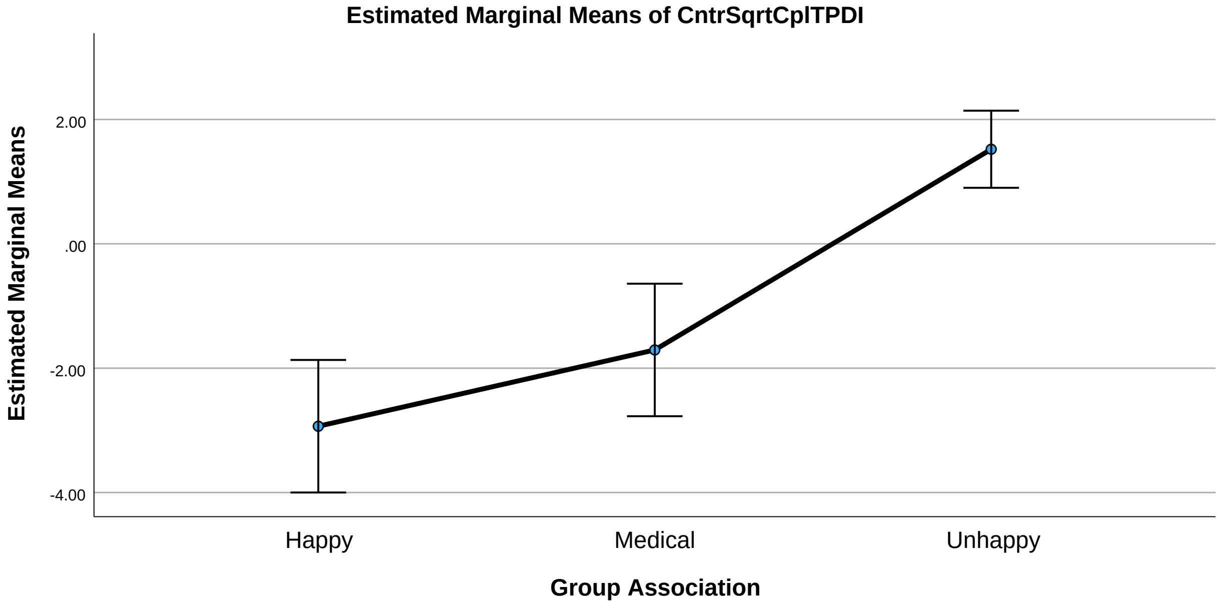

Covariates appearing in the model are evaluated at the following values: HYears of Education = 15.1188, HAge of Participant = 42.6535, WYears of Education = 11.5248, WAge of Participant = 41.2574

Error bars: 95% CI

Univariate Analysis of Variance

Notes

Output Created		20-DEC-2023 11:15:16
Comments		
Input	Data	C:\Users\jdwir\OneDrive\Marvin Research\DATA Sets\DyadicData_WholeLot_122 (2023).sav
	Active Dataset	DataSet1
	Filter	<none>
	Weight	<none>
	Split File	<none>
	N of Rows in Working Data File	101
Missing Value Handling	Definition of Missing	User-defined missing values are treated as missing.
	Cases Used	Statistics are based on all cases with valid data for all variables in the model.
Syntax		UNIANOVA CplQScore BY GroupAssociation WITH YearsEd Age YearsEdWif AgeWif<br>/METHOD=SSTYPE(3)<br>/INTERCEPT=INCLUDE<br>/PLOT=PROFILE (GroupAssociation) TYPE=LINE ERRORBAR=CI MEANREFERENCE=NO YAXIS=AUTO<br>/EMMEANS=TABLES (GroupAssociation) WITH (YearsEd=MEAN Age=MEAN YearsEdWif=MEAN AgeWif=MEAN) COMPARE<br>ADJ(BONFERRONI)<br>/PRINT ETASQ DESCRIPTIVE HOMOGENEITY OPOWER<br>/CRITERIA=ALPHA(.05)<br>/DESIGN=YearsEd Age ...
Resources	Processor Time	00:00:00.14
	Elapsed Time	00:00:00.13

Between-Subjects Factors

		Value Label	N
Group Association	1.00	Medical	20
	2.00	Unhappy	61
	3.00	Happy	20

Descriptive Statistics

Dependent Variable: CplQScore

Group Association	Mean	Std. Deviation	N
Medical	.5090	.26872	20
Unhappy	.2566	.24944	61
Happy	.5905	.27148	20
Total	.3727	.29408	101

Levene's Test of Equality of Error Variances[a]

Dependent Variable: CplQScore

F	df1	df2	Sig.
.708	2	98	.495

Tests the null hypothesis that the error variance of the dependent variable is equal across groups.

a. Design: Intercept + YearsEd + Age + YearsEdWif + AgeWif + ...

Tests of Between-Subjects Effects

Dependent Variable: CplQScore

Source	Type III Sum of Squares	df	Mean Square	F	Sig.	Partial Eta Squared
Corrected Model	2.670[a]	6	.445	6.997	<.001	.309
Intercept	1.060	1	1.060	16.669	<.001	.151
YearsEd	.318	1	.318	5.001	.028	.051
Age	.133	1	.133	2.085	.152	.022
YearsEdWif	.056	1	.056	.878	.351	.009
AgeWif	.043	1	.043	.679	.412	.007
GroupAssociation	1.012	2	.506	7.954	<.001	.145
Error	5.978	94	.064			
Total	22.676	101				
Corrected Total	8.649	100				

Tests of Between-Subjects Effects

Dependent Variable: CplQScore

Source	Noncent. Parameter	Observed Power[b]
Corrected Model	41.982	.999
Intercept	16.669	.981
YearsEd	5.001	.600
Age	2.085	.298
YearsEdWif	.878	.153
AgeWif	.679	.129
GroupAssociation	15.908	.950
Error		
Total		
Corrected Total		

a. R Squared = .309 (Adjusted R Squared = .265)

b. Computed using alpha = .05

Estimated Marginal Means

Group Association

Estimates

Dependent Variable: CplQScore

Group Association	Mean	Std. Error	95% Confidence Interval Lower Bound	95% Confidence Interval Upper Bound
Medical	.506[a]	.065	.377	.635
Unhappy	.263[a]	.038	.188	.338
Happy	.574[a]	.065	.446	.703

a. Covariates appearing in the model are evaluated at the following values: HYears of Education = 15.1188, HAge of Participant = 42.6535, WYears of Education = 11.5248, WAge of Participant = ...

Pairwise Comparisons

Dependent Variable: CplQScore

(I) Group Association	(J) Group Association	Mean Difference (I-J)	Std. Error	Sig.[b]	95% Confidence Interval for ...[b] Lower Bound
Medical	Unhappy	.243*	.082	.012	.043
	Happy	-.068	.083	1.000	-.271
Unhappy	Medical	-.243*	.082	.012	-.444
	Happy	-.311*	.082	<.001	-.512
Happy	Medical	.068	.083	1.000	-.134
	Unhappy	.311*	.082	<.001	.111

Pairwise Comparisons

Dependent Variable: CplQScore

(I) Group Association	(J) Group Association	95% Confidence Interval for ...[b] Upper Bound
Medical	Unhappy	.444
	Happy	.134
Unhappy	Medical	-.043
	Happy	-.111
Happy	Medical	.271
	Unhappy	.512

Based on estimated marginal means

*. The mean difference is significant at the .05 level.

b. Adjustment for multiple comparisons: Bonferroni.

Univariate Tests

Dependent Variable: CplQScore

	Sum of Squares	df	Mean Square	F	Sig.	Partial Eta Squared
Contrast	1.012	2	.506	7.954	<.001	.145
Error	5.978	94	.064			

Univariate Tests

Dependent Variable: CplQScore

	Noncent. Parameter	Observed Power[a]
Contrast	15.908	.950
Error		

The F tests the effect of Group Association. This test is based on the linearly independent pairwise comparisons among the estimated marginal means.

a. Computed using alpha = .05

Profile Plots

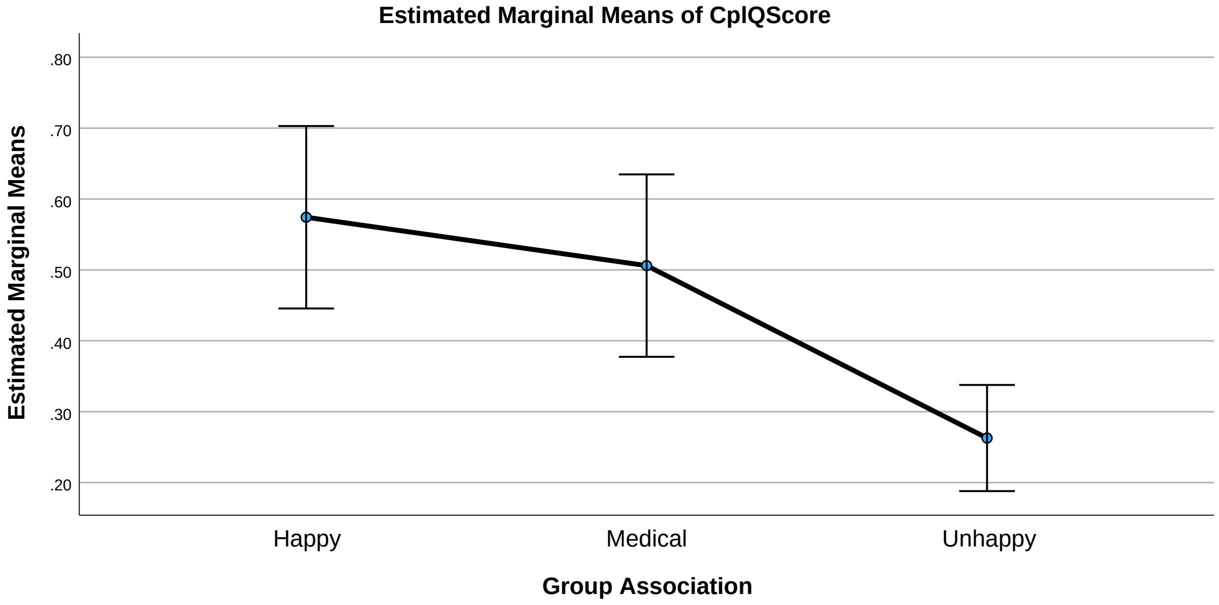

Covariates appearing in the model are evaluated at the following values: HYears of Education = 15.1188, HAge of Participant = 42.6535, WYears of Education = 11.5248, WAge of Participant = 41.2574

Error bars: 95% CI
