# Supplementary material for: Assessing self–other agreement and dyadic adjustment in marital dyads
Source: Front Psychol. 2024 Nov 15;15:1363165. doi: 10.3389/fpsyg.2024.1363165 (PMC11604461; doi:10.3389/fpsyg.2024.1363165)
Supplement: Supplementary file 6 [file Data_Sheet_6.PDF]

Univariate Analysis of Variance

Notes

Output Created		16-SEP-2023 15:17:04
Comments		
Input	Data	C: \Users\jdwir\OneDrive\Marvin Research\DATA Sets\DyadicData_WholeLot_122 (2023).sav
	Active Dataset	DataSet1
	Filter	<none>
	Weight	<none>
	Split File	<none>
	N of Rows in Working Data File	101
Missing Value Handling	Definition of Missing	User-defined missing values are treated as missing.
	Cases Used	Statistics are based on all cases with valid data for all variables in the model.

Notes

Syntax		UNIANOVA Level_CplDASODA BY GroupAssociation WITH Ethnicity YearsEd Age EthnicityWif YearsEdWif AgeWif<br>/METHOD=SSTYPE(3)<br>/INTERCEPT=INCLUDE<br>/PLOT=PROFILE (GroupAssociation) TYPE=LINE ERRORBAR=CI MEANREFERENCE=NO YAXIS=AUTO<br>/EMMEANS=TABLES (GroupAssociation) WITH (Ethnicity=MEAN YearsEd=MEAN Age=MEAN EthnicityWif=MEAN YearsEdWif=MEAN AgeWif=MEAN) COMPARE ADJ (BONFERRONI)<br>/PRINT ETASQ DESCRIPTIVE HOMOGENEITY OPOWER<br>/CRITERIA=ALPHA(.05)<br>/DESIGN=Ethnicity YearsEd Age EthnicityWif ...
Resources	Processor Time	00:00:00.37
	Elapsed Time	00:00:00.18

Between-Subjects Factors

		Value Label	N
Group Association	1.00	Medical	20
	2.00	Unhappy	61
	3.00	Happy	20

Descriptive Statistics

Dependent Variable: Level_CplDASODA

Group Association	Mean	Std. Deviation	N
Medical	2.0500	.75915	20
Unhappy	1.3934	.49257	61
Happy	2.5500	.51042	20
Total	1.7525	.72672	101

Levene's Test of Equality of Error Variances[a]

Dependent Variable: Level_CplDASODA

F	df1	df2	Sig.
.654	2	98	.522

Tests the null hypothesis that the error variance of the dependent variable is equal across groups.

a. Design: Intercept + Ethnicity + YearsEd + Age + EthnicityWif + YearsEdWif + AgeWif + GroupAssociation

Tests of Between-Subjects Effects

Dependent Variable: Level_CplDASODA

Source	Type III Sum of Squares	df	Mean Square	F	Sig.	Partial Eta Squared
Corrected Model	23.867[a]	8	2.983	9.483	<.001	.452
Intercept	3.676	1	3.676	11.685	<.001	.113
Ethnicity	.024	1	.024	.077	.782	.001
YearsEd	.001	1	.001	.004	.951	.000
Age	.441	1	.441	1.402	.239	.015
EthnicityWif	.009	1	.009	.028	.867	.000
YearsEdWif	.203	1	.203	.644	.424	.007
AgeWif	.016	1	.016	.051	.823	.001
GroupAssociation	14.184	2	7.092	22.542	<.001	.329
Error	28.945	92	.315			
Total	363.000	101				
Corrected Total	52.812	100				

Tests of Between-Subjects Effects

Dependent Variable: Level_CplDASODA

Source	Noncent. Parameter	Observed Power[b]
Corrected Model	75.861	1.000
Intercept	11.685	.923
Ethnicity	.077	.059
YearsEd	.004	.050
Age	1.402	.216
EthnicityWif	.028	.053
YearsEdWif	.644	.125
AgeWif	.051	.056
GroupAssociation	45.083	1.000
Error		
Total		
Corrected Total		

a. R Squared = .452 (Adjusted R Squared = .404)

b. Computed using alpha = .05

Estimated Marginal Means

Group Association

Estimates

Dependent Variable: Level_CplDASODA

Group Association	Mean	Std. Error	95% Confidence Interval	
			Lower Bound	Upper Bound
Medical	2.026[a]	.147	1.735	2.317
Unhappy	1.379[a]	.085	1.211	1.547
Happy	2.618[a]	.145	2.330	2.906

a. Covariates appearing in the model are evaluated at the following values: HHEthnicity = 4.0891, HYears of Education = 15.1188, HAge of Participant = 42.6535, WEthnicity = 3.8020, WYears of Education = 11.5248, WAge of Participant = 41.2574.

Pairwise Comparisons

Dependent Variable: Level_CplDASODA

(I) Group Association	(J) Group Association	Mean Difference (I-J)	Std. Error	Sig.[b]	95% Confidence Interval for [b]... Lower Bound
Medical	Unhappy	.647*	.187	.002	.192
	Happy	-.592*	.187	.006	-1.049
Unhappy	Medical	-.647*	.187	.002	-1.103
	Happy	-1.239*	.185	<.001	-1.690
Happy	Medical	.592*	.187	.006	.135
	Unhappy	1.239*	.185	<.001	.789

Pairwise Comparisons

Dependent Variable: Level_CplDASODA

(I) Group Association	(J) Group Association	95% Confidence Interval for [b]... Upper Bound
Medical	Unhappy	1.103
	Happy	-.135
Unhappy	Medical	-.192
	Happy	-.789
Happy	Medical	1.049
	Unhappy	1.690

Based on estimated marginal means

*. The mean difference is significant at the .05 level.

b. Adjustment for multiple comparisons: Bonferroni.

Univariate Tests

Dependent Variable: Level_CplDASODA

	Sum of Squares	df	Mean Square	F	Sig.	Partial Eta Squared
Contrast	14.184	2	7.092	22.542	<.001	.329
Error	28.945	92	.315			

Univariate Tests

Dependent Variable: Level_CplDASODA

	Noncent. Parameter	Observed Power[a]
Contrast	45.083	1.000
Error		

The F tests the effect of Group Association. This test is based on the linearly independent pairwise comparisons among the estimated marginal means.

a. Computed using alpha = .05

Profile Plots

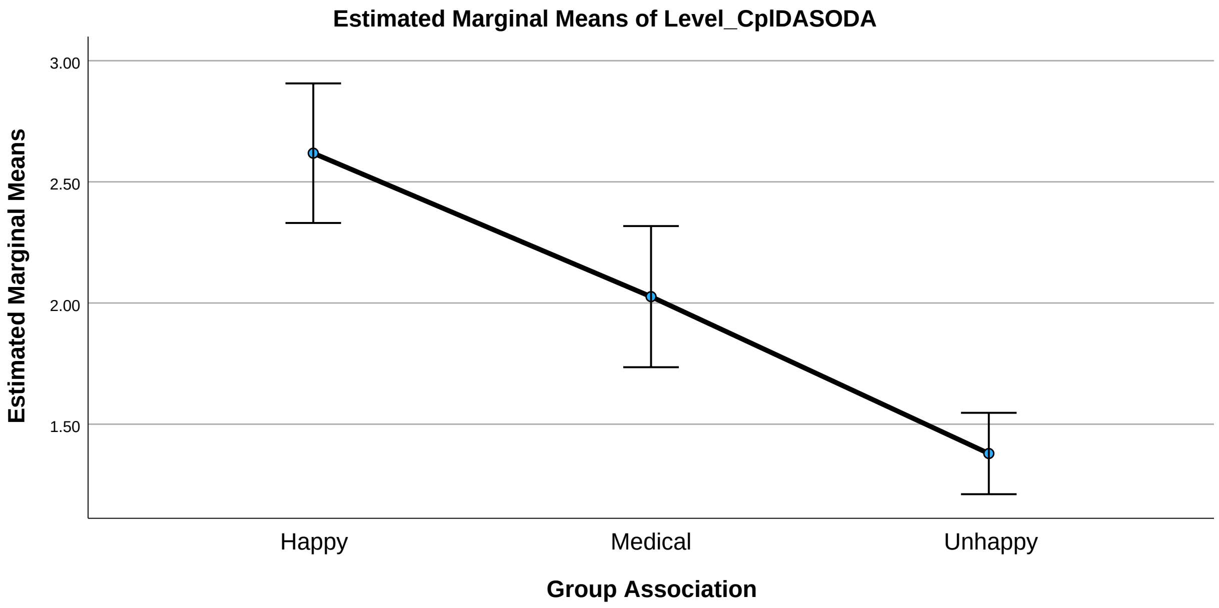

Covariates appearing in the model are evaluated at the following values: HHEthnicity = 4.0891, HYears of Education = 15.1188, HAge of Participant = 42.6535, WEthnicity = 3.8020, WYears of Education = 11.5248, WAge of Participant = 41.2574

Error bars: 95% CI
