# Supplementary material for: Assessing self–other agreement and dyadic adjustment in marital dyads
Source: Front Psychol. 2024 Nov 15;15:1363165. doi: 10.3389/fpsyg.2024.1363165 (PMC11604461; doi:10.3389/fpsyg.2024.1363165)
Supplement: Supplementary file 7 [file Data_Sheet_7.PDF]

Regression

Descriptive Statistics

	Mean	Std. Deviation	N
Level_CplDASODA	1.7525	.72672	101
Level_CplQscore	1.7624	.73687	101

Correlations

		Level_CplDASODA	Level_CplQscore
Pearson Correlation	Level_CplDASODA	1.000	.337
	Level_CplQscore	.337	1.000
Sig. (1-tailed)	Level_CplDASODA	.	<.001
	Level_CplQscore	.000	.
N	Level_CplDASODA	101	101
	Level_CplQscore	101	101

Variables Entered/Removed[a]

Model	Variables Entered	Variables Removed	Method
1	Level_CplQscore[b]	.	Enter

a. Dependent Variable: Level_CplDASODA

b. All requested variables entered.

Model Summary

Model	R	R Square	Adjusted R Square	Std. Error of the Estimate	Change Statistics		
					R Square Change	F Change	df1
1	.337[a]	.114	.105	.68759	.114	12.705	1

Model Summary

Model	Change Statistics	
	df2	Sig. F Change
1	99	<.001

a. Predictors: (Constant), Level_CplQscore

ANOVA[a]

Model		Sum of Squares	df	Mean Square	F	Sig.
1	Regression	6.007	1	6.007	12.705	<.001[b]
	Residual	46.805	99	.473		
	Total	52.812	100			

a. Dependent Variable: Level_CplDASODA

b. Predictors: (Constant), Level_CplQscore

Coefficients[a]

Model		Unstandardized Coefficients		Standardized Coefficients	t	Sig.
		B	Std. Error	Beta		
1	(Constant)	1.166	.178		6.548	<.001
	Level_CplQscore	.333	.093	.337	3.564	<.001

Coefficients[a]

Model		95.0% Confidence Interval for B		Collinearity Statistics	
		Lower Bound	Upper Bound	Tolerance	VIF
1	(Constant)	.813	1.520		
	Level_CplQscore	.147	.518	1.000	1.000

a. Dependent Variable: Level_CplDASODA

Coefficient Correlations[a]

Model			Level_CplQscore
1	Correlations	Level_CplQscore	1.000
	Covariances	Level_CplQscore	.009

a. Dependent Variable: Level_CplDASODA

Collinearity Diagnostics[a]

Model	Dimension	Eigenvalue	Condition Index	Variance Proportions	
				(Constant)	Level_CplQscore
1	1	1.923	1.000	.04	.04
	2	.077	5.007	.96	.96

a. Dependent Variable: Level_CplDASODA
